# LncRNA SNHG26 promotes gastric cancer progression and metastasis by inducing c-Myc protein translation and an energy metabolism positive feedback loop

**DOI:** 10.1038/s41419-024-06607-8

**Published:** 2024-03-29

**Authors:** Zhen-Hua Wu, Yi-Xuan Wang, Jun-Jiao Song, Li-Qin Zhao, Yu-Jia Zhai, Yan-Fang Liu, Wei-Jian Guo

**Affiliations:** 1 Department of Medical Oncology, Fudan University Shanghai Cancer Center, Shanghai, 200032 China; 2 Department of Oncology, Shanghai Medical College, Fudan University, Shanghai, 200032 China; 3 Fudan University Shanghai Cancer Center and Institutes of Biomedical Sciences, Shanghai, 200032 China; 4 Department of Oncology, Ruijin Hospital, Shanghai Jiao Tong University School of Medicine, Shanghai, 200025 China

**Keywords:** Gastric cancer, Long non-coding RNAs, Ribosome

## Abstract

Metastasis is a bottleneck in cancer treatment. Studies have shown the pivotal roles of long noncoding RNAs (lncRNAs) in regulating cancer metastasis; however, our understanding of lncRNAs in gastric cancer (GC) remains limited. RNA-seq was performed on metastasis-inclined GC tissues to uncover metastasis-associated lncRNAs, revealing upregulated small nucleolar RNA host gene 26 (SNHG26) expression, which predicted poor GC patient prognosis. Functional experiments revealed that SNHG26 promoted cellular epithelial–mesenchymal transition and proliferation in vitro and in vivo. Mechanistically, SNHG26 was found to interact with nucleolin (NCL), thereby modulating c-Myc expression by increasing its translation, and in turn promoting energy metabolism via hexokinase 2 (HK2), which facilitates GC malignancy. The increase in energy metabolism supplies sufficient energy to promote c-Myc translation and expression, forming a positive feedback loop. In addition, metabolic and translation inhibitors can block this loop, thus inhibiting cell proliferation and mobility, indicating potential therapeutic prospects in GC.

## Introduction

Gastric cancer (GC) is the fifth most commonly diagnosed tumor in humans, with over 1 million estimated new cases diagnosed annually [1, 2]. GC is the fourth most common cause of cancer-related death due to its high metastatic frequency, with over 60% of patients presenting with local or distant metastasis at diagnosis [3, 4]. Despite substantial progress in the development of molecular targeted drugs and immunotherapy, the 5-year survival rate of GC patients remains unsatisfactory due to metastasis [5, 6]. Thus, investigations into the mechanisms underlying GC metastasis are urgently required for the development of new therapeutic approaches for GC.

Long noncoding RNAs (lncRNAs) exceed 200 nucleotides and have limited protein-coding capacities [7, 8]. LncRNAs are known to be widely expressed and play pivotal roles in gene regulation [9]. LncRNAs perform diverse functions through interactions with RNA, DNA, and proteins [10]. Numerous studies have demonstrated that aberrant expression of lncRNAs is associated with cancer cell behaviors [11]. For example, lncRNA C1RL-AS1 promotes GC proliferation and metastasis via the AKT/β-catenin pathway [12]. The lncRNA BDNF-AS/WDR5/FBXW7 axis mediates ferroptosis in GC peritoneal metastasis by regulating VDAC3 ubiquitination [13]. The lncRNA TNFRSF10A-AS1 promotes GC progression by binding with MPZL1 [14]. However, due to the large number of lncRNAs and their complicated mechanisms, our understanding of lncRNAs in GC remains limited.

Lymph node metastasis (LNM) is an important determinant of disease progression in GC patients [3, 4]. Several studies have shown that cancer cells in lymph nodes participate in dissemination to distant organs [15]. Recently, numerous studies have described the association between aberrant lncRNA expression and progression in human cancers [9, 16]. However, little is known about the role of lncRNAs in LNM in GC. Early gastric cancer (EGC) is defined as the invasion of the submucosa by cancer cells regardless of the metastasis status. We reasoned that GC diagnosed at the T1 stage with lymph node metastasis (pT_1_N_+_M_0_) might demonstrate high metastatic potential and malignant behavior.

In this study, RNA-seq was performed on six paired EGC tissues (pT_1_N_+_M_0_), screening out lncRNA small nucleolar RNA host gene 26 (SNHG26). A loss- and gain-of-function analysis revealed that SNHG26 promoted EMT and proliferation in vitro and in vivo. Mechanistically, SNHG26 could promote c-Myc translation in an IRES-dependent manner via NCL. In addition, SNHG26 was found to mediate glycolysis and energy metabolism through the c-Myc/hexokinase 2 (HK2) pathway, promoting GC cell proliferation and migration. The increase in glycolytic metabolism replenishes energy and promotes mRNA translation, forming a positive feedback loop. The above findings reveal that lncRNA SNHG26 is a potential therapeutic target in GC.

## Materials and methods

### Clinical samples and ethical statement

A total of 93 paired human GC and adjacent noncancerous tissues and six paired EGC tissues with LNM were obtained from the biobank of Fudan University Shanghai Cancer Center (FUSCC). Each patient underwent gastrectomy from 2008 – 2015 at FUSCC, and none received chemotherapy or radiation before surgery. The use of all clinical specimens in our study was approved by the ethics committee of FUSCC. The clinicopathological characteristics of the patients are shown in Table 1 and described according to the 7^th^ version of the American Joint Committee on Cancer Staging System (AJCC).

**Table 1:** Relationships between SNHG26 expression and clinicopathological features of GC patients.

Parameter	No. of patients	SNHG26 (low)	SNHG26 (high)	*P*-value
Age (years)				0.2969
<60	39	22	17	
≥60	54	24	30	
Gender				0.6411
Male	69	33	36	
Female	24	13	11	
Tumor size				
≤5 cm	51	28	23	0.2996
>5 cm	42	18	24	
Histologic grade				0.5362
Moderately / Moderately-Poorly differentiated	46	21	25	
Poorly differentiated	47	25	22	
pT stage				
T1-3	35	21	14	0.1370
T4	58	25	33	
pN stage				**0**.0299*
N0	16	12	4	
N1-3	77	34	43	
pTNM stage				0.1798
I + II	28	17	11	
III	65	29	36	

^*^P < 0.05 was considered significant.

Bold values represents *P* value <0.05.

### RNA sequencing

Tumor and paired adjacent noncancerous tissues from six EGC patients diagnosed with LNM were analyzed by transcriptomic sequencing (RNA-seq). Total RNA from these samples was extracted using the TRIzol total RNA extraction protocol (Invitrogen). RNA quality and quantity were assessed on Agilent DNA/RNA 6000 chips (Agilent Technologies) and Qubit (Life Technologies) before next-generation sequencing library preparation. RNA-seq libraries were constructed using the TruSeq RNA Sample Prep Kit (Illumina) and subjected to paired-end (2 × 150 bp) sequencing on an Illumina HiSeq 3000 platform. The raw RNA-Seq reads in FASTQ format were aligned to the human reference genome hg38 using HISAT [17]. Name-sorted and indexed BAM files were subsequently generated by SAMtools (v1.8-47) [18]. FeatureCount [19] was used to generate transcript count files. The count matrix was then obtained using DESeq2 [20]. We used fragments per kilobase million (FPKM) to evaluate the expression levels of individual genes by normalizing the gene length using the count matrix. The R package limma [21] was used to identify differentially expressed genes (DEGs) using normalized read counts. The above data have been submitted to the GEO database (GSE192468).

### RNA extraction, reverse-transcription PCR, and quantitative real-time PCR (qRT-PCR)

RNA extraction, reverse transcription PCR, and qRT-PCR were conducted as previously described [12, 22]. The primer sequences are listed in the Supplementary Table 1.

### Cell lines, culture, and reagents

Human GC cell lines (MKN-45, MKN-28, MGC-803, AGS, and HGC-27), the normal gastric epithelial cell line GES-1, and HEK293T cells were acquired from Shanghai Cell Bank Type Culture Collection Committee (CBTCCC, Shanghai, China) and maintained at 37 °C with 5% CO_2_. All cells were cultured in Dulbecco’s modified Eagle’s medium (DMEM) (HyClone, Logan, USA) supplemented with 10% fetal bovine serum (FBS), 100 U/ml penicillin, and 100 μg/ml streptomycin (Invitrogen). Stable cell lines were treated with selected inhibitors, maintained at 37 °C, and harvested at the indicated time points for subsequent analysis. All the cell lines were recently authenticated by short tandem repeat analysis. The proteasome inhibitor cycloheximide (CHX) and the HK2 inhibitor 2-deoxy-d-glucose (2-DG) were purchased from Sigma‒Aldrich (Germany). Ribosomal RNA synthesis inhibitor CX-5461 and oligomycin were purchased from MedChemExpress Company (USA).

### RNA interference

Smart silencers targeting the lncRNA SNHG26 and siRNAs targeting NCL were synthesized by RiboBio (Guangzhou, China). GC cells were seeded in 6-well plates, and after cell adherence, the smart silencer and siRNAs were transfected at a final concentration of 50 nM using Lipofectamine RNAiMAX (Invitrogen). The indicated cells were harvested for protein extraction and proliferation, migration, and invasion assays after transfection for 48 h.

### 5′ and 3′ rapid amplification of cDNA ends (RACE) assay

A 5′ and 3′ RACE assay was utilized to determine the transcriptional initiation and termination sites of lncRNAs using a SMARTer RACE cDNA Amplification kit (Clontech, California, USA) following the manufacturer’s instructions. The specific sequences of the primers used for the 5′ and 3′ RACE analysis are provided in the Supplementary Table 1.

### Subcellular fractionation

For nuclear/cytoplasmic fractionation, GC cells (1 × 10^7^ of each) were collected and suspended in cell fraction buffer according to the manufacturer’s instructions (Ambion^TM^, PARIS^TM^). Then, RNA was extracted, and qRT-PCR was performed. β-Actin and 18 S served as the cytoplasmic endogenous control, and U1 small nuclear RNA served as the nuclear endogenous control.

### RNA fluorescence in situ hybridization (FISH)

Probes targeting lncRNAs in GC cells were designed and synthesized by RiboBio (Guangzhou, China). The assay was performed using a Ribo^TM^ Fluorescent In Situ Hybridization Kit (Ribobio Company, China). Briefly, cells were plated onto glass slides (Merck Millipore), followed by fixation with 4% paraformaldehyde for 15 min at room temperature. Then, the cells were permeabilized with 0.5% Triton X-100 for 5 min at 4 °C. Subsequently, the cells were blocked with prehybridization buffer for 30 min at 37 °C. Furthermore, the cells were incubated in hybridization buffer with a FISH probe at 37 °C in the dark overnight. The cells were then washed three times with Buffer I (4 × SSC with 0.1% Tween-20), once with Wash Buffer II (2 × SSC), incubated with Wash Buffer III (1 × SSC) at 42 °C in the dark for 5 min, and washed once with PBS at room temperature. Subsequently, the cells were stained with Prolong Gold Antifade reagent with DAPI (Thermo Fisher Scientific).

The Cy3-labeled probes targeting SNHG26 and RNA FISH in GC and adjacent normal tissues were synthesized and performed by Servicebio Company (Wuhan, China). Images were acquired using a confocal microscope.

### Cell proliferation and colony formation assays

CCK-8 and colony formation assays were performed as previously described [22]. For the EdU (5-ethynyl-2′-deoxyuridine) assay, the cells were detached and seeded in a 12-well plate. The following day, the cells were washed with PBS and incubated with the corresponding concentration of EdU buffer for 2 h. After incubation, the cells were washed with PBS for 5 min twice and then incubated with 4% paraformaldehyde for 15 min at room temperature, followed by 0.25% Triton X-100 in PBS and staining with click reaction solution (Beyotime, China). Images were acquired at 40 × using a Leica microscope and deposited for further analysis.

### Transwell assays

The migration and invasion assays were performed as described previously [12, 22].

### RNA pull-down assay

LncRNAs were transcribed and labeled with Biotin RNA Labeling Mix (Roche, USA) and purified with an RNeasy Mini Kit (QIAGEN, USA). Subsequently, the biotinylated RNAs were incubated with protein supernatant isolated from GC cells at 4 °C for 1 h. Then, 30 μl streptavidin beads (Invitrogen, USA) were added to the complexes, and the samples were incubated at 4 °C overnight. The proteins were then collected, separated, and silver-stained. Specific bands were isolated and analyzed by mass spectrometry (Shanghai Applied Protein Technology, Shanghai, China). The primers for the lncRNA and its deletion fragments used for in vitro transcription are provided in Supplementary Table 1.

### RNA immunoprecipitation (RIP) assay

The RIP assay was performed with the Magna RIP RNA-binding Protein Immunoprecipitation kit (Millipore, USA) according to the manufacturer’s instructions. In brief, cells were pelleted and lysed with RIP lysis buffer on ice for 1 h. Then, the supernatants were harvested by centrifugation at 12000 × g for 10 min at 4 °C. Subsequently, 5 µg of primary antibody and the corresponding IgG control were added to the supernatant. After incubation at 4 °C overnight, 30 µl of protein G beads were mixed with the complexes. After 2.5 h of incubation at 4 °C, the above complexes were washed with RIP lysis buffer five times each. The RNA was extracted with TRIzol reagent (Invitrogen).

### Chromatin immunoprecipitation (ChIP) assay

A ChIP assay was performed according to the manufacturer’s instructions (Millipore Merck, Germany). Briefly, SNHG26 vector and OE cells were incubated with 37% formaldehyde in PBS for 10 min at room temperature. Formaldehyde was quenched by adding 2 mL glycine, and the cells were harvested by centrifugation and washed three times in cold PBS. Then, the cell extracts were centrifuged in Buffer A (5 mM PIPES, 85 mM KCl, 0.5% NP-40, protease inhibitor), and the cell precipitates were resuspended in Buffer B (100 mM Tris-Cl, 1% sodium dodecyl sulfate (SDS), 10 mM EDTA, protease inhibitor). Ultrasonic shearing of chromatin was performed on wet ice. Aliquots containing 500 ng of DNA were diluted 10-fold with IP buffer (0.01% SDS, 1.1% Triton X-100, 1.2 mM EDTA, 16.7 mM Tris-Cl, 167 mM NaCl and protease inhibitor) and incubated with primary antibody Nucleolin overnight at 4 °C. Samples were incubated with protein A- or protein G-linked agarose beads for 2–4 h at 4 °C. The agarose–antibody/chromatin complexes were washed with wash buffer and then washed twice with TE buffer. Chromatin was eluted with elution buffer, 8 μl of 5 M NaCl was added, and the samples were incubated at 65 °C for 4–5 h or overnight to allow DNA-protein reverse cross-linking. One microliter of Ribonuclease A was then added, and the samples were incubated at 37 °C for 30 min. Subsequently, 4 μl 0.5 EDTA, 8 μl 1 M Tris-HCl, and 1 μl proteinase K was added, and the samples were incubated at 45 °C for 1–2 h. DNA was purified using a centrifugal column and analyzed.

### Western blotting

Western blotting was performed as described previously [12]. The primary antibodies were listed in Supplementary Table 2. The secondary antibodies were as follows: goat anti-rabbit or anti-mouse IgG (1:10000 each; Jackson ImmunoResearch Laboratories).

### Co-immunoprecipitation (Co-IP) assay

For Co-IP, cells were cultured to confluence in a 15 cm dish, followed by PBS washes. An IP cell lysis buffer containing the cocktail and optional phosphatase inhibitors was added, and the cells were incubated on ice. Antibody-magnetic bead complexes were prepared with two sets of beads, each washed thrice. After incubating cell lysates with these complexes, supernatants were collected, mixed with loading buffer, and heated for total protein analysis. Excess antibody was removed using a magnetic stand, and the remaining supernatant was incubated overnight with IgG and target antibody-bead complexes. After washing and elution, the samples were analyzed by western blotting.

### Polysome profiling

Cells were incubated with 100 μg/ml cycloheximide (CHX) per 10 cm dish at 37 °C for 10 min. Then, the cells were collected and lysed on ice with lysis buffer for 3 min. After centrifugation at 12000 × g for 5 min at 4 °C, the supernatant was loaded onto a 15/50% (w/v) sucrose gradient solution prepared in lysis buffer. Centrifugation was performed at 4 °C for 2 h 40 min at 27,400 × g (Beckman, SW40 rotor). The RNA content was then fractioned and measured using a Gradient Station (BioCamp) coupled with a UV monitor (Bio-Rad) and a fraction collector (FC203B, Gilson). After fractionation, RNA was extracted using TRIzol and analyzed by qRT-PCR.

### Glycolytic process analysis and ATP assays

Lactate Colorimetric Assay Kits (Biovision, USA) and Glucose Uptake Colorimetric Assay Kits (Biovision, USA) were used to detect the glycolytic process according to the manufacturer’s instructions. An ATP assay kit (Beyotime, China) was used per the manufacturer’s directions to detect ATP levels in GC cell lines after the indicated treatment.

### Dual luciferase assays

HEK293T cells (1 × 10^4^) were seeded in 24-well plates. The cells were transfected with 0.5 µg each of pRF reporter plasmid and pRmF reporter plasmid using transfection reagent jetPRIMER and incubated for 48 h. The luciferase activity was measured using a dual luciferase reporter gene assay kit (Promega, USA) according to the manufacturer’s instructions to obtain the firefly and Renilla luminescence sites.

### O-propargyl-puromycin (OP-Puro) Click-iT protein synthesis assay

The OP-Puro Click-iT protein synthesis assay was performed according to the manufacturer’s instructions (Thermo Fisher Scientific, USA). In brief, after 2 days of incubation, the cells were incubated for 30 min using 20 μM Click-iT OPP reagent. After washing with cold PBS, the cells were fixed with 4% formaldehyde for 20 min and subsequently permeabilized with 0.5% Triton X-100 (Beyotime, China) for 15 min. After washing twice in PBS, the cells were incubated in a reaction solution containing 0.25% Alexa Fluor picolylazide for 30 min at room temperature in the dark, washed three times in PBS, and visualized by laser confocal microscopy.

### Puromycin labeling

Protein synthesis was assessed using puromycin labeling (SUnSET technique) as previously described [23]. Puromycin (10 μg/mL, Thermo Fisher Scientific) was added to the cells for in vitro labeling. Cells were incubated with puromycin for 15 min, transferred to fresh complete medium, and incubated for 1 h. Cell lysates were collected for immunoblotting, and immunoblot protein loading was normalized using the cell count.

### In vivo assay

Male BALB/c nude mice were purchased and raised in the Experimental Animal Center of Fudan University Shanghai Cancer Center. A total of 1 × 10^7^ MKN-28 cells stably expressing control vector or SNHG26-OE were injected subcutaneously into each nude mouse (4 weeks old, six per group) to evaluate the effect of SNHG26 on tumorigenesis. The general behavior of the mice was monitored, and tumor volumes were measured every 3 days for 28 days. Moreover, after 2 weeks, the mice were randomly assigned to the indicated groups and received subsequent inhibitors. The investigator was blinded to the group allocation of the animals during the experiment. At the end of the experiments, the mice were sacrificed, and tumor volumes were calculated by the following equation: volume = (length × width^2^ × 0.5). To further explore the effect of SNHG26 on metastasis in vivo, 3 × 10^6^/200 μl MKN-28 cells (stably expressing the control vector or SNHG26-OE) were injected into the tail vein, and the mice (4 weeks old, six per group) were sacrificed after 8 weeks. The number of metastatic foci in the lung was determined by tissue section HE staining. No data were excluded from the analysis.

### Immunohistochemistry (IHC)

Immunohistochemistry was performed as previously reported [22]. The sections were incubated with the primary antibody against E-cadherin (1:500, Cell Signaling Technology).

### Targeted metabolomics

A total of 1 × 10^6^ GC cell deposits were collected and divided into vector control and SNHG26 overexpression groups. The samples were quickly frozen in liquid nitrogen and stored in a –80 °C freezer. The samples were later thawed on ice. The cells were then resuspended in 100 μl of ultrapure water to extract the cell precipitate. Fifty microliters of the cell suspension was added to 200 μl of pure methanol extract (–20 °C, precooled) and mixed well. The samples were then centrifuged at 12000 r/min for 10 min at 4 °C and then allowed to stand in the freezer at –20 °C for 30 min. The supernatant was collected after centrifugation at 12000 r/min for 10 min at 4 °C. Ultra performance liquid chromatography (Waters, US) and tandem mass spectrometry (SCIES, US) were used for data acquisition.

### Statistical analyses

Each experiment was performed in triplicate, and representative data from one experiment are displayed. All values are presented as the mean ± standard error of the mean (SEM). Student’s *t*-test was used to determine the significance of differences between groups. Pearson’s chi-square test was used to assess correlations between lncRNA expression and clinicopathological features. Overall survival was analyzed using the Kaplan‒Meier method with a log-rank test. *P-*values < 0.05 were considered to indicate significance.

## Results

### Identification and characterization of lncRNA SNHG26 in GC

**Fig. 1:**
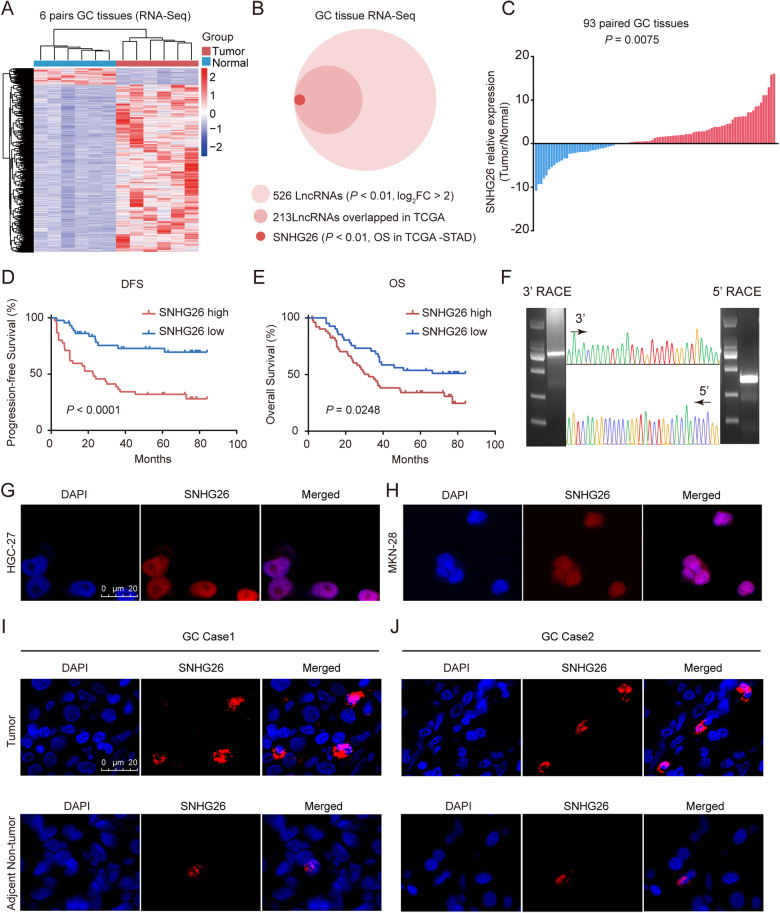
Identification and characterization of lncRNA SNHG26 in GC. **A** Heat-map representation of RNA-sequencing in six paired EGC tissues with LNM. **B** Screening and identification of SNHG26 by RNA-seq. **C**. RNA levels of SNHG26 in 93 pairs of GC and adjacent normal tissues by qPCR. **D, E** Kaplan–Meier analysis of the correlation between SNHG26 RNA levels and disease-free survival and overall survival times in 93 GC patients. **F** Representative images of PCR products from 3’ RACE and 5’ RACE. **G, H** RNA FISH images showing the distributions of SNHG26 in HGC-27 and MKN-28 cells. Bar: 20 μM. **I, J** RNA FISH images showing the distributions of SNHG26 in GC and adjacent normal tissues. Bar: 20 μM.

RNA-seq was conducted on six paired GC tissues with LNM pathology to uncover differentially expressed lncRNAs in GC (Fig. 1A, Supplementary Fig. S1A). A total of 526 lncRNAs were significantly upregulated in GC tissues versus adjacent nontumor tissues (*p* < 0.01, log2FC > 2) (Fig. 1B). Overall survival data in TCGA STAD were available for 213 of the 526 lncRNAs, and 80 were correlated with poorer survival times. Among these 80 lncRNAs, the lncRNA SNHG26 ranked first in terms of *P-*value. Thus, we selected SNHG26 as the candidate lncRNA for further study. Moreover, an mRNA GSEA enrichment analysis of the RNA-seq results revealed that multiple biological processes, including EMT, were involved in GC malignancy (Supplementary Fig. S1B).

To confirm the correlation between SNHG26 expression and patient outcomes, we conducted qPCR to measure SNHG26 expression in 93 paired GC tissues obtained from the FUSCC. Consistently, SNHG26 levels were dramatically increased in GC tissue compared with those in adjacent tissue (Fig. 1C). Subsequent survival analysis revealed that patients with higher expression of SNHG26 had shorter disease-free survival (DFS) times (*p* < 0.0001) and overall survival (OS) times (*p* = 0.0248) than those with lower expression (Fig. 1D, E). TCGA STAD also showed that SNHG26 indicated poor survival times (*p* = 0.00075) (Supplementary Fig. 1C). In addition, the clinicopathological feature analysis (Table 1) showed that SNHG26 expression correlated with pN status (*P* = 0.0299). A trend difference in pT (*P* = 0.1370) and pTNM (*P* = 0.1798) stage was also observed.

SNHG26 is located on chromosome 7, and five annotated transcripts are available in the National Center for Biotechnology Information database (NCBI) (Supplementary Fig. S1D). The 5′ and 3′ RACE results (Fig. 1F) revealed that the 1,106 bp SNHG26 was the predominant transcript. The sequence of the full-length SNHG26 is shown in Supplementary Fig. S1E. The Coding Potential Calculator (CPC) (http://cpc.gao-lab.org), Coding Potential Assessment Tool (CPAT) (http://lilab.research.bcm.edu/cpat/index.php), and PhyloCSF codon substitution frequency analysis predicted that SNHG26 was a noncoding RNA (Supplementary Fig. S1F–H). Moreover, RNA FISH of the GC cell lines HGC-27 and MKN-28 (Fig. 1G, H), GC tumor and adjacent nontumor tissues (Fig. 1I, J), and the subcellular fractionation of HGC-27 (Supplementary Fig. S1I) suggested that SNHG26 was mainly distributed in the nucleus. Consistently, the LncATLAS (http://lncatlas.crg.eu) results also demonstrated that SNHG26 was mainly located in the nucleus (Supplementary Fig. S1J). Overall, the above results suggest that SNHG26 is mainly located in the nucleus and might play an oncogenic role in GC.

### SNHG26 promotes proliferation and metastasis in vitro and in vivo in GC

**Fig. 2:**
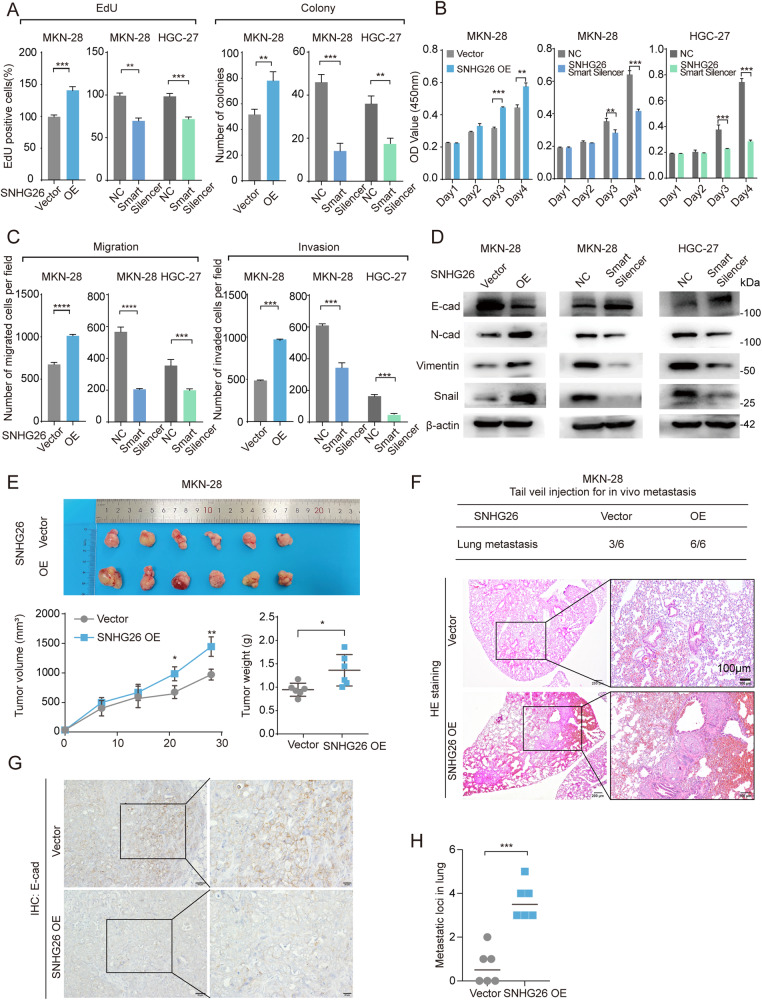
SNHG26 promotes proliferation and metastasis capabilities of GC in vitro and in vivo. **A** EdU and colony formation assays in SNHG26-overexpressing MKN-28 cells or in SNHG26-knockdown MKN-28 and HGC-27 cells. **B** CCK-8 assays in SNHG26-overexpressing MKN-28 cells or SNHG26-knockdown MKN-28 and HGC-27 cells. **C** Migration and invasion assays in SNHG26-overexpressing MKN-28 cells or SNHG26-knockdown MKN-28 and HGC-27 cells. **D** E-cadherin, N-cadherin, Vimentin, and Snail measured by western blotting after modulating SNHG26 expression. **E** Representative images of tumors from nude mice subcutaneously injected with MKN-28 cells infected with lentivirus expressing SNHG26 or control lentivirus. **F**, **H** HE staining of sections with metastatic nodules in lung samples obtained from nude mice after injection with MKN-28 cells infected with lentivirus expressing SNHG26 (**F**) Bars: 200 μM; 100 μM. Statistical analysis of the metastatic foci in the lung detected by HE staining (H). **G** Representative images of IHC staining with E-cadherin after SNHG26 overexpression. Bars: 20 μM; 50 μM. Values are expressed as the mean ± SEM, *n* = 6. All data are from three independent experiments. The data are presented as the mean ± SD values (*n* ≥ 3). *P < 0.05; **P< 0.01; ***P < 0.001; ****P < 0.0001.

To investigate the biological function of SNHG26 in GC, we first detected the RNA level of SNHG26 in different gastric cancer cell lines. SNHG26 was expressed at higher levels in HGC-27 cells and at lower levels in MKN-28 cells (Supplementary Fig. 2A). Then, MKN-28 cells were infected with a lentivirus harboring the SNHG26 full-length sequence to generate a stably overexpressing cell line, and lncRNA smart silencers were applied to the MKN-28 and HGC-27 cell lines to reduce SNHG26 RNA levels (Supplementary Fig. 2B). Overexpressing SNHG26 accelerated cell growth and colony formation in MKN-28 cells, whereas knockdown dramatically inhibited the proliferation of MKN-28 and HGC-27 cells (Fig. 2A, B, Supplementary Fig. 2C–F). Additionally, ectopic expression of SNHG26 significantly altered the migration and invasion capacities of MKN-28 cells, while knockdown reduced MKN-28 and HGC-27 cell mobility (Fig. 2C, Supplementary Fig. 2G). Moreover, EMT-associated markers were detected by western blotting after modulating the status of SNHG26. Overexpression of SNHG26 increased N-cadherin (N-cad), Vimentin (VIM), and Snail protein expression levels but reduced E-cadherin (E-cad) expression, and knockdown produced opposite results (Fig. 2D), indicating the potential role of SNHG26 in regulating EMT.

To confirm the oncogenic role of SNHG26 in vivo, we first generated a subcutaneous tumor model by injecting SNHG26-overexpressing MKN-28 cells subcutaneously into nude mice. After 4 weeks, the mice were sacrificed, and the tumors were weighed and measured. Tumors in the SNHG26 overexpression group were larger than those in the control group, indicating that SNHG26 could promote tumor growth (Fig. 2E). Next, we injected SNHG26-overexpressing MKN-28 cells into the tail vein of nude mice to assess metastatic capacities. After 8 weeks, the mice were sacrificed, and metastatic loci in the lung were quantified. Furthermore, HE staining revealed that the lungs of the SNHG26-overexpressing group demonstrated a significantly higher number of metastatic loci than those of the control group (Fig. 2F, H). Moreover, IHC of the subcutaneous tumor showed that E-cad expression was decreased after SNHG26 overexpression (Fig. 2G). Overall, the in vitro and in vivo results suggest that SNHG26 promotes GC proliferation and metastasis.

### Identification of SNHG26-associated proteins

**Fig. 3:**
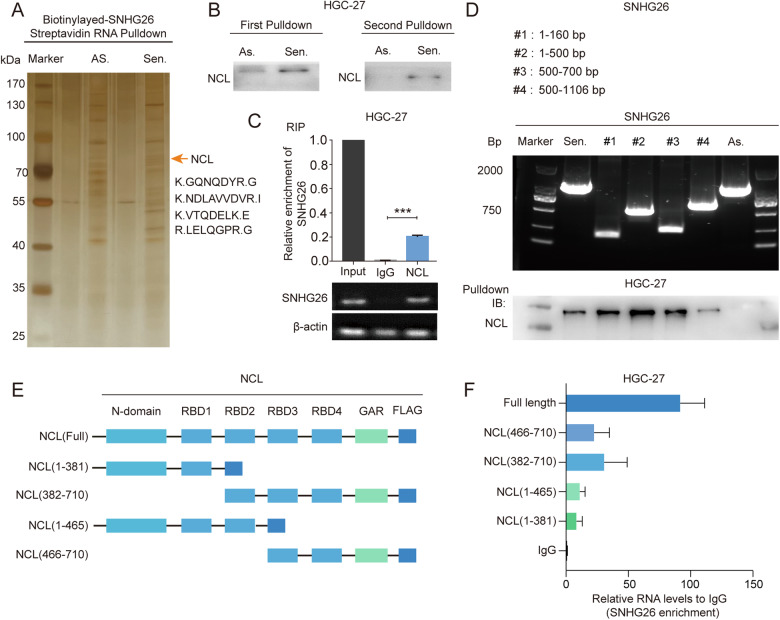
Identification of SNHG26-associated proteins. **A**. RNA pull-down assay showing the proteins that bind with biotinylated SNHG26 in vitro by silver staining. **B**. Immunoblotting for specific associations of NCL with SNHG26 from RNA pull-down assays. **C**. RIP assays were performed using antibodies against NCL, and qPCR was used to detect SNHG26 enrichment. **D**. Immunoblotting for NCL in samples pulled down with full-length biotinylated-SNHG26 or truncated RNA motifs. **E**, **F**. Deletion mapping to identify the domains of NCL that bind to SNHG26. RIP analysis for SNHG26 enrichment in cells transiently transfected with full-length or truncated FLAG-tagged constructs.

As discussed above, lncRNAs exert their biological function through diverse mechanisms, including interactions with DNA, RNA, and proteins. An RNA pull-down assay was conducted to identify proteins that bind to SNHG26. After SDS‒PAGE and silver staining, a specific differential band at the position of 70-100 kd of the sense group was excised and subjected to mass spectrometry (Fig. 3A). The independent western blot analysis revealed nucleolin (NCL) as one of the binding targets of SNHG26 (Fig. 3B). Further RIP assays confirmed that NCL directly interacted with SNHG26 (Fig. 3C). In addition, we performed deletion-mapping analysis to map the precise binding regions between SNHG26 and NCL due to the secondary structure (Supplementary Fig. 3A). As shown in Fig. 3D, the in vitro RNA pull-down indicated that the 1–500 bp sequence of SNHG26 was crucial for its interaction with NCL. Regarding the protein domain (Fig. 3E, Supplementary Fig. 3B), SNHG26 interacted with the 382–710 amino acid (aa) region of NCL (Fig. 3F). Overall, these results suggest that SNHG26 likely exerts its function by binding with NCL.

### SNHG26 promotes mRNA translation in GC

NCL is a nucleocytoplasmic protein involved in many biological processes, including ribosomal assembly and rRNA processing, indicating that NCL might be linked with ribosomal biogenesis [24, 25]. Intriguingly, we found that NCL could modulate the expression of ribosomal proteins, such as RPS3 and RPL4 (Supplementary Fig. 4A). The qPCR results showed that ribosomal prerequisite RNA (47 S rRNA) expression was upregulated after SNHG26 overexpression and vice versa when silencing SNHG26 (Supplementary Fig. 4B). Additionally, NCL might interact with RPS3 and RPL4 (www.string .com) (Supplementary Fig. 4C). Co-IP revealed that NCL interacted with RPS3 and RPL4 in the SNHG26 vector and overexpressing MKN-28 cells (Supplementary Fig. 4D). Moreover, a recent study demonstrated that NCL binds to the promoter and coding regions of rDNA and stimulates rDNA transcription [26]. Given the above findings, we used ChIP analysis to verify the distribution of NCL on the rDNA of GC cells at specific rDNA promoters and coding regions. SNHG26 overexpression increased NCL occupancy in the rDNA coding region (H8, H13; Supplementary Fig. 4E). This finding suggests that SNHG26 promotes the binding of NCL to the rDNA promoter and coding regions to enhance transcription.

**Fig. 4:**
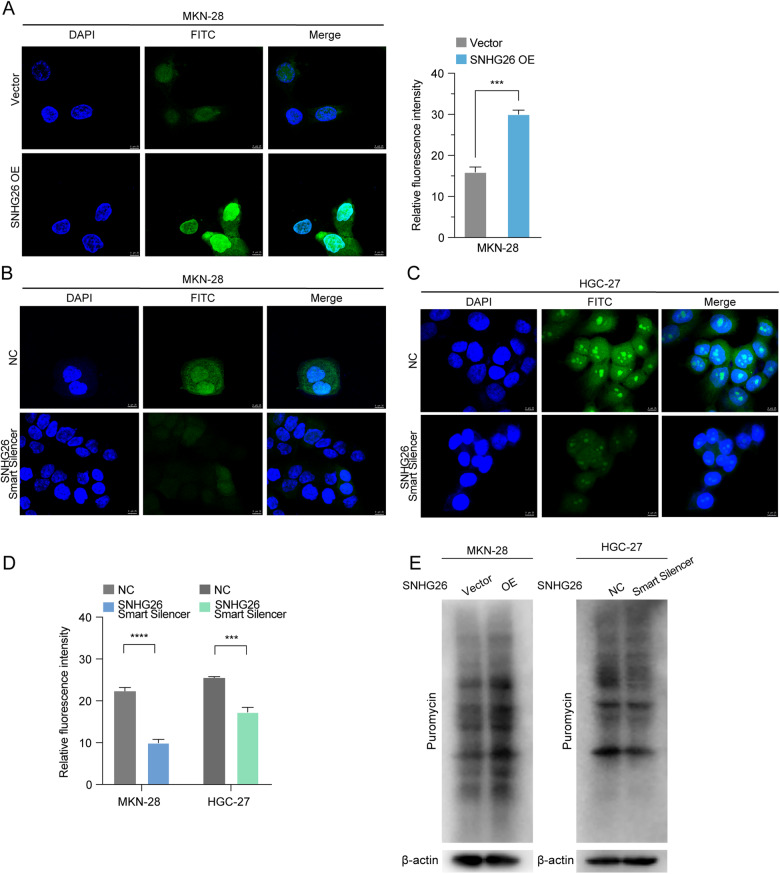
SNHG26 promotes protein synthesis in GC. **A**–**C** Protein synthesis was measured in MKN-28 and HGC-27 GC cell lines with SNHG26 overexpressed or knocked down (OPP, green; DAPI, blue). Scale bars = 25 μM. **D** Quantification of Alexa 488 fluorescence intensity. **E** Western blotting was performed to assess protein synthesis in MKN-28 and HGC-27 cell lines overexpressing or knocking down SNHG26 treated with puromycin labeling, followed by detection with puromycin antibody. All data are from three independent experiments. The data are presented as the mean ± SD values (*n* = 3). *P <0.05; **P <0.01; ***P <0.001; ****P<0.0001.

Dysregulated ribosomal biogenesis is usually associated with protein translation, indicating that SNHG26 might promote protein translation via NCL. We then performed an OPP assay to detect active protein synthesis in GC cells to test this hypothesis. Significantly increased protein synthesis was observed in SNHG26-overexpressing MKN-28 cells, whereas protein synthesis was reduced in HGC-27 and MKN-28 cells after SNHG26 knockdown (Fig. 4A–D). In addition, we performed SUnSET assays to detect the puromycin-labeled polypeptides and evaluate global protein synthesis. Consistent with the OPP assay results, global protein synthesis changes were detected after ectopic expression of SNHG26 (Fig. 4E). Overall, the above results suggest that SNHG26 promotes mRNA translation and protein synthesis in GC.

### LncRNA SNHG26 promotes the proliferation and migration of GC cells by stimulating c-Myc translation in an NCL-dependent manner

**Fig. 5:**
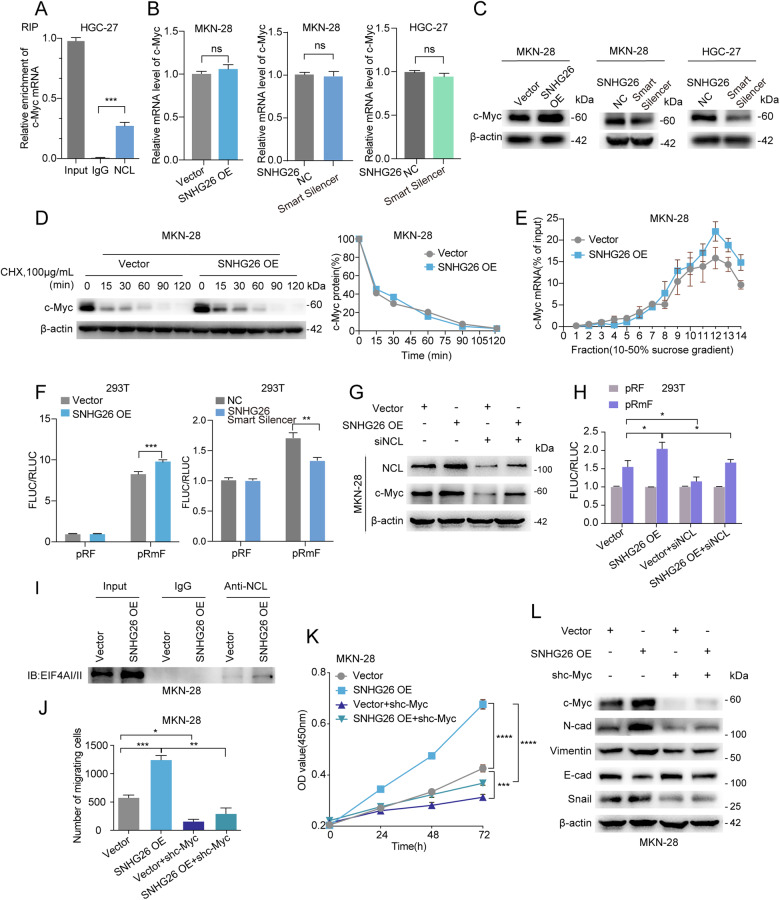
LncRNA SNHG26 promotes the proliferation and migration of GC cells by stimulating c-Myc translation in an NCL-dependent manner. **A** c-Myc mRNA was highly enriched in the RNAs enriched by anti-Flag NCL RIP by RT-qPCR in HGC-27 cells. **B** mRNA expression of c-Myc was detected by qPCR after modulating SNHG26 status in MKN-28 and HGC-27 GC cells. **C** Protein expression of c-Myc was examined by western blotting after modulating SNHG26 status in MKN-28 and HGC-27 GC cells. **D** A CHX (100 µg/ml) chase experiment showed no significant change in the half-life of c-Myc protein in MKN-28 cells overexpressing SNHG26. **E** Percentage of c-Myc mRNAs measured by qPCR in each fraction collected from the polysome profiling after overexpressing SNHG26 in MKN-28 cells. **F** Expression of pRF with pRmF of firefly or Renilla luciferase in 293 T cells after overexpression or knockdown of SNHG26. **G** Western blotting showing that the increases in the levels of NCL and c-Myc were significantly reversed after transfection of siNCL into SNHG26-overexpressed MKN-28 cells. **H** The expression of firefly versus Renilla luciferase was evaluated after transfection of cells stably expressing the empty vector, SNHG26 gene, with pRF, pRmF reporter plasmid, and NC, siNCL small interfering RNA. **I** A Co-IP assay verified the presence of interactions between NCL and EIF4AI/II. **J** MKN-28 cells overexpressing SNHG26 were transfected with shc-Myc or shNC for 48 h. Cell malignant metastasis was determined by Transwell assays. **K** MKN-28 cells overexpressing SNHG26 were transfected with shc-Myc or shNC for 48 h. Cell proliferation was determined by CCK8. **L** Western blot showing that the increases in N-cadherin, Vimentin, Snail, and c-Myc levels were significantly reversed after transfection of shc-Myc plasmid into SNHG26-overexpressing cells for 48 h. In contrast, the decrease in E-cadherin levels was significantly reversed. All data are from three independent experiments. The data are presented as the mean ± SD values (*n*≥ 3). *P < 0.05; **P < 0.01; ***P < 0.001; ****P < 0.0001.

Interestingly, previous studies have shown that NCL can directly bind with the promoter region of c-Myc and transcriptionally regulate its expression [27]. We first performed RIP assays to investigate whether SNHG26 binding to NCL also transcriptionally promotes c-Myc expression. As shown in Fig. 5A, our experimental results revealed that NCL can bind to c-Myc mRNA. However, lncRNA SNHG26 significantly regulated c-Myc expression at the protein level but not at the mRNA level (Fig. 5B, C), suggesting that SNHG26 does not transcriptionally upregulate c-Myc expression, different from the reported mechanism by which NCL upregulates c-Myc expression. Moreover, after treatment with CHX over time, the half-life of c-Myc did not change after SNHG26 overexpression (Fig. 5D). The above results indicate that SNHG26 regulates c-Myc not through transcription but possibly through a translational mechanism.

To determine whether SNHG26 regulates c-Myc translation, we conducted polysome profiling in SNHG26-overexpressing cells. The qPCR results showed that upregulated SNHG26 significantly increased the translation of c-Myc mRNA in MKN-28 cells (Fig. 5E). To revalidate the experimental results of polysome profiling, we used bicistronic reporters to investigate the 5’ cap- and internal ribosomal entry site (IRES)-dependent translation efficiencies. The luciferase reporter results indicated that, compared to control cells, SNHG26-overexpressing cells demonstrated increased IRES-dependent translation activity, as indicated by increased firefly luciferase activity (Fig. 5F). It has been reported that NCL can self-regulate its expression by binding to the c-Myc promoter [28]. To explore whether NCL can increase its transcription by binding to the promoter of c-Myc in GC, we conducted a ChIP experiment; however, NCL did not bind to c-Myc (Supplementary Fig. 5A), consistent with the literature. It has been shown that typical posttranscriptional Cap-dependent translation is achieved by an increase in RNA binding to ribosomes [29]. In contrast, IRES-dependent translation does not result in an increase in RNA binding and may be achieved by increasing the rate of bound ribosome assembly. We first performed western blotting experiments to demonstrate whether SNHG26 promotes c-Myc protein translation dependent on NCL. NCL knockdown in SNHG26-overexpressing GC cells significantly downregulated c-Myc expression (Fig. 5G). Luciferase reporter gene experiments showed that NCL knockdown reduced the IRES-dependent translational activity of c-Myc caused by SHNG26 overexpression (Fig. 5H). Additionally, Co-IP showed that NCL interacted with the translation initiation factor EIF4AI/II (Fig. 5I). The above results suggest that SNHG26 recruits NCL to the IRES site of c-Myc mRNA and facilitates NCL recruitment of the transcription initiation factor EIF4AI/II with ribosomes to initiate IRES-dependent Myc translation. To determine whether function is exerted through c-Myc, we silenced c-Myc in GC cells overexpressing SNHG26 and controls. CCK8 and Transwell assays as well as western blotting showed that downregulation of c-Myc expression could reverse the alteration of proliferation, migration, and EMT marker expression in GC cells caused by SNHG26 overexpression (Fig. 5J–L, Supplementary Fig. 5B).

CX-5461 is a recently developed inhibitor of ribosomal RNA synthesis that selectively inhibits Pol I-driven transcription, DNA replication, and protein translation [30]. To determine whether CX-5461 inhibits the proliferation and migration ability of GC cells, we performed CCK-8 and Transwell assays in MKN-28 cells, revealing that CX-5461 treatment decreased the proliferation and migration abilities of GC cells compared to those observed in the control group (Supplementary Fig. 5C, D). The subsequent OPP and luciferase reporter gene assays showed that the increase in translation of SNHG26 action was similarly inhibited by treatment with CX-5461 (Supplementary Fig. 5E–G). Overall, CX-5461 inhibited SNHG26-mediated protein translation, thus suppressing the migration and proliferation capabilities of GC cells.

### SNHG26 regulates energy metabolism through the c-Myc/HK2 pathway, facilitating GC proliferation and migration

**Fig. 6:**
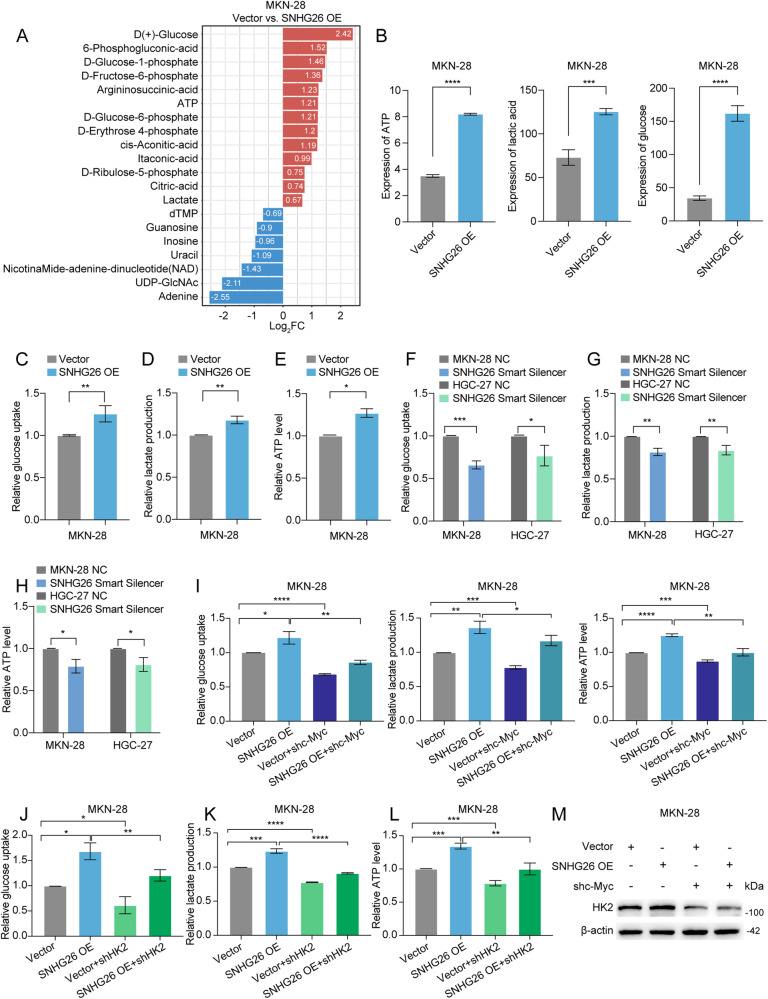
SNHG26 regulates energy metabolism through the c-Myc/HK2 pathway. **A** Differentially abundant metabolite bar chart. **B** Differentially abundant metabolite histograms with horizontal coordinates indicating group and vertical coordinates indicating expression. **C**–**E** Changes in glucose uptake, lactate production, and ATP levels in MKN-28 cells overexpressing SNHG26. **F**–**H** Changes in glucose uptake, lactate production, and ATP levels in MKN-28 and HGC-27 cells with SNHG26 knockdown. **I** Glucose uptake, lactate production, and ATP levels were enhanced after lncRNA SNHG26 overexpression, while changes in metabolic levels were reversed by silencing c-Myc. **J**–**L**. The increases in glucose uptake, lactate production, and ATP levels were significantly reversed after transfection of the shHK2 plasmid into SNHG26-overexpressing cells. **M** Western blotting showing that the increase in HK2 levels was significantly reversed after transfection of the shc-Myc plasmid into SNHG26-overexpressing cells for 48 h. All data are from three independent experiments. The data are presented as the mean ± SD values (*n* ≥ 3). *P < 0.05; **P < 0.01; ***P < 0.001; ****P < 0.0001.

Recently, several studies have indicated that dysregulated energy metabolism supports cancer cell growth and facilitates metastasis [31]. Previous studies have shown a link between c-Myc regulation and cellular metabolism, proliferation, and metastasis [32]. To explore whether SNHG26 demonstrates the above mechanism, we first performed targeted metabolomics in Metware Biotech Company (Wuhan, China). In this assay, 42 metabolites were tested for differentially abundant metabolite screening by fold change ≥1.5 or ≤0.67 as metabolic thresholds. Multiple differentially abundant metabolites were identified, thirteen upregulated and seven downregulated (Fig. 6A). There was an upregulation in the levels of metabolites of interest, such as glucose, lactate, and ATP (Fig. 6B). Given the targeted metabolomics results, we verified the changing levels of glucose, lactate, and ATP. Glucose uptake, lactate production, and ATP levels were dramatically increased after overexpressing SNHG26 in MKN-28 cells and decreased when SNHG26 was knocked down in HGC-27 cells (Fig. 6C–H). Next, we silenced c-Myc in GC cells overexpressing SNHG26, significantly decreasing glucose uptake, lactate production, and ATP levels (Fig. 6I).

**Fig. 7:**
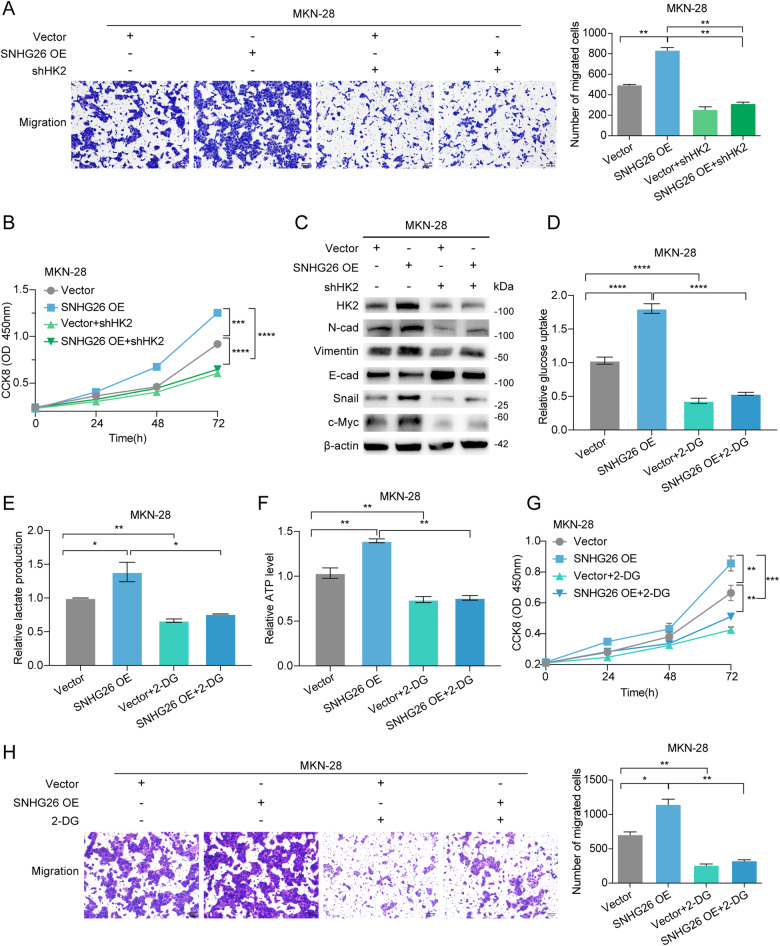
SNHG26 regulates energy metabolism through the c-Myc/HK2 pathway, facilitating GC proliferation and migration. **A** Transwell assays showed that the increased migration of SNHG26-overexpressing cells was significantly reversed after transfection with the shHK2 plasmid. Scale bars = 100 μM. **B** The CCK-8 assay showed that the increased proliferation of SNHG26-overexpressing cells was significantly reversed after transfection with the shHK2 plasmid. **C** Western blotting showing that the increases in HK2, N-cadherin, Vimentin, Snail, and c-Myc levels were significantly reversed after transfection of the shHK2 plasmid into SNHG26-overexpressing cells for 48 h. In contrast, the decrease in E-cadherin levels was significantly reversed. **D**–**F** Glucose uptake, lactate production, and ATP levels were significantly decreased after the addition of 2-DG (5 mM) in control cells and SNHG26-overexpressing cells. **G** MKN-28 cells overexpressing SNHG26 were treated with 2-DG (5 mM). Cell proliferation was determined by CCK8 assay. **H** MKN-28 cells overexpressing SNHG26 were treated with 2-DG (5 mM). Cell malignant metastasis was determined by Transwell assay. Scale bars = 100 μM. All data are from three independent experiments. The data are presented as the mean ± SD values (*n* ≥ 3). *P < 0.05; **P < 0.01; ***P < 0.001; ****P < 0.0001.

To explore which glycolysis-related proteins can be regulated by SNHG26, we first measured the expression of glycolysis-related proteins after modulating SNHG26. As shown in Supplementary Fig. 6A, HK2 but not GLUT1, LDHA, LDHB, ALDOA, or ALDOB was significantly upregulated after overexpressing SNHG26. We knocked down HK2 in SNHG26-overexpressing MKN-28 cells, and as expected, the detected glucose uptake, lactate production, and ATP levels were inhibited (Fig. 6J–L). We silenced the expression of c-Myc, and the expression of HK2 was correspondingly downregulated (Fig. 6M). Then, we detected the proliferation and migration capacities and EMT-associated markers of MKN-28 SNHG26-overexpressing cells after HK2 knockdown. The Transwell and CCK8 assays as well as western blotting analyses showed that the migration and proliferation abilities and EMT marker expression patterns were reversed after silencing HK2 expression (Fig. 7A–C). Interestingly, c-Myc expression was also downregulated after HK2 knockdown (Fig. 7C).

To investigate whether SNHG26 promotes GC cell proliferation and migration through the HK2-mediated glycolytic pathway, we added the HK2 inhibitor 2-DG to SNHG26-overexpressing cells to inhibit glycolysis, and treatment with 2-DG abolished the SNHG26-induced increase in glucose uptake, lactate production, and ATP production (Fig. 7D–F). We next assessed the effect of 2-DG on the proliferation and migratory capacity of GC cells. 2-DG significantly reduced the proliferation and migration capabilities of SNHG26-OE cells (Fig. 7G, H). We also performed ATP compensation experiments, revealing that the proliferation and metastatic capacities of the cells were significantly improved by back-supplementing ATP in the presence of inhibited ATP levels (Supplementary Fig. 7A, B). This finding suggests that SNHG26 regulates energy metabolism through the c-Myc/HK2 pathway and promotes GC proliferation and migration.

### SNHG26 promotes energy production through regulation of the c-Myc/HK2 pathway, and positive feedback promotes the translation and expression of c-Myc

As we had previously found that c-Myc expression was downregulated after HK2 knockdown, we hypothesized that SNHG26 promotes energy production by regulating the c-Myc/HK2 pathway, which positively feeds back to promote c-Myc translation and expression. To test this hypothesis, we applied an OPP assay and luciferase reporter gene assay, and the results suggested that the overall protein translation and translation abilities of the c-Myc IRES were inhibited after the addition of 2-DG in SNHG26-overexpressing MKN-28 GC and control cells, respectively (Supplementary Fig. 7C–E). Western blotting indicated that the addition of 2-DG inhibited c-Myc expression, while the addition of ATP partially restored its expression (Supplementary Fig. 7F). When we added oligomycin, an ATP inhibitor, overexpression of SNHG26 caused an increase in c-Myc expression that was suppressed by oligomycin (Supplementary Fig. 7G). The above results suggest that SNHG26 promotes energy production by regulating the c-Myc/HK2 pathway, and positive feedback promotes the translation and expression of c-Myc.

### The combination of a translation inhibitor (CX-5461) and a metabolic inhibitor (2-DG) had significant therapeutic effects in vivo and vitro

**Fig. 8:**
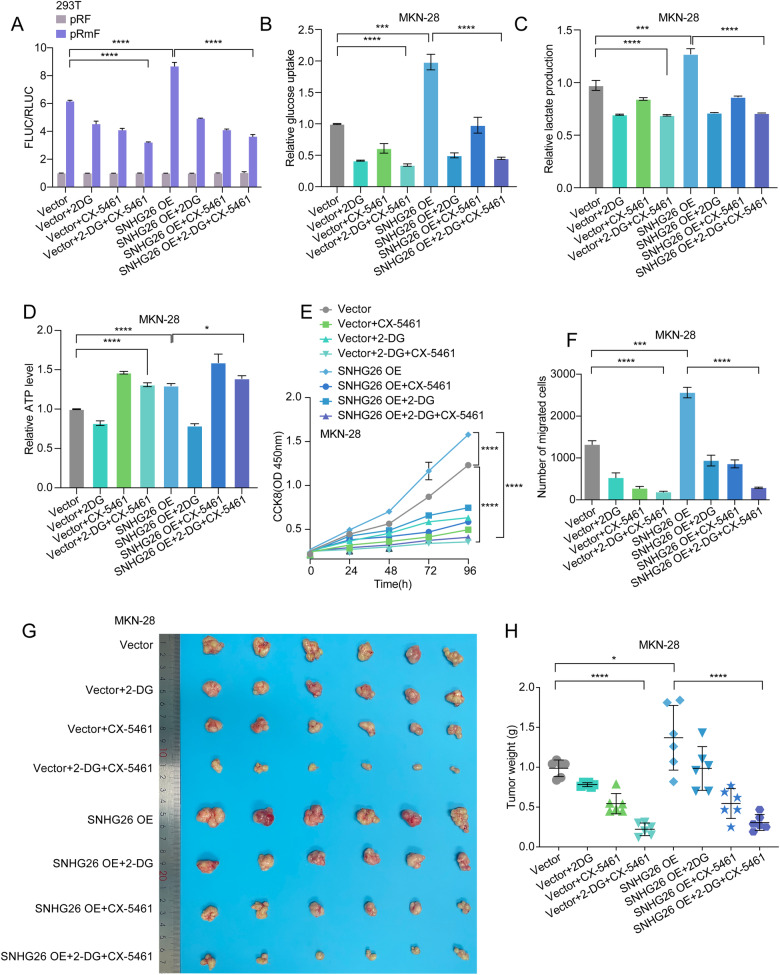
The combination of a translation inhibitor (CX-5461) and a metabolic inhibitor (2-DG) had significant therapeutic effects in vivo and vitro. **A** 293 T cells stably expressing the empty vector and SNHG26 gene were transfected with pRF and pRmF reporter plasmids with or without 2-DG and CX-5461 and assessed for firefly and Renilla luciferase expression. **B**–**D**. Changes in glucose uptake, lactate production, and ATP levels in MKN-28 cells were examined after treatment with 2-DG alone, CX-5461 alone, and combined treatment with 2-DG and CX-5461. **E**, **F** The proliferation and migration abilities of MKN-28 cells were analyzed by CCK-8 and Transwell assays after treatment with 2-DG alone, CX-5461 alone, and the combination of 2-DG and CX-5461. **G** Nude mice were injected with control or SNHG26-overexpressing cells and treated with blank (PBS), 2-DG (500 mg/kg), CX-5461 (40 mg/kg), or both drugs in combination after 1 week. Images of xenograft tumors in nude mice sacrificed after 4 weeks (*n* = 6). **H** Tumor weights of nude mice treated as described above. All data are from three independent experiments. The data are presented as the mean ± SD values (*n* ≥ 3). *P < 0.05; **P < 0.01; ***P < 0.001; ****P < 0.0001.

We next demonstrated the effects of treatment with the metabolic inhibitor 2-DG alone, the ribosome inhibitor CX-5461 alone, and the combination of 2-DG and CX-5461 on translation and metabolic levels in HEK293T and MKN-28 cells and found that the combination treatment group significantly reduced translation levels, glucose uptake, and lactate production in GC cells (Fig. 8A–C, Supplementary Fig. 8A, B). Interestingly, the level of ATP was higher in the CX-5461 group than in the control group (Fig. 8D). We speculate that capacity consumption may decrease after translation inhibition. Although we found that treatment with each drug alone inhibited GC cell proliferation and migration compared to controls, 2-DG combined with CX-5461 was significantly more potent than single-drug treatment. The inhibitory effect of the combination of 2-DG and CX-5461 in the SNHG26 overexpression group was more pronounced than that in the control group (Fig. 8E, F, Supplementary Fig. 8C). Moreover, we generated a subcutaneous tumor model by injecting control cells and SNHG26-overexpressing MKN-28 cells subcutaneously into nude mice. After 2 weeks, the mice were randomly assigned into four groups, one receiving blank saline, one receiving the ribosomal inhibitor CX-5461, one receiving the metabolic inhibitor 2-DG, and the last receiving a ribosomal inhibitor in combination with a metabolic inhibitor. After 4 weeks, the mice were sacrificed, and the tumors were weighed and measured. As indicated, injection of MKN-28 cells overexpressing SNHG26 significantly promoted tumor progression. Mice in the groups receiving ribosomal or metabolic inhibitors had smaller tumors than those in the control group, the tumor volume in the combination treatment group was smaller than that in the other three groups (Fig. 8G, H, Supplementary Fig. 8D), suggesting combination of the two inhibitors could increase the antitumor effect. Overall, our results suggest that SNHG26/NCL/c-Myc could be a potential biomarker of GC and therapeutic target.

## Discussion

**Fig. 9:**
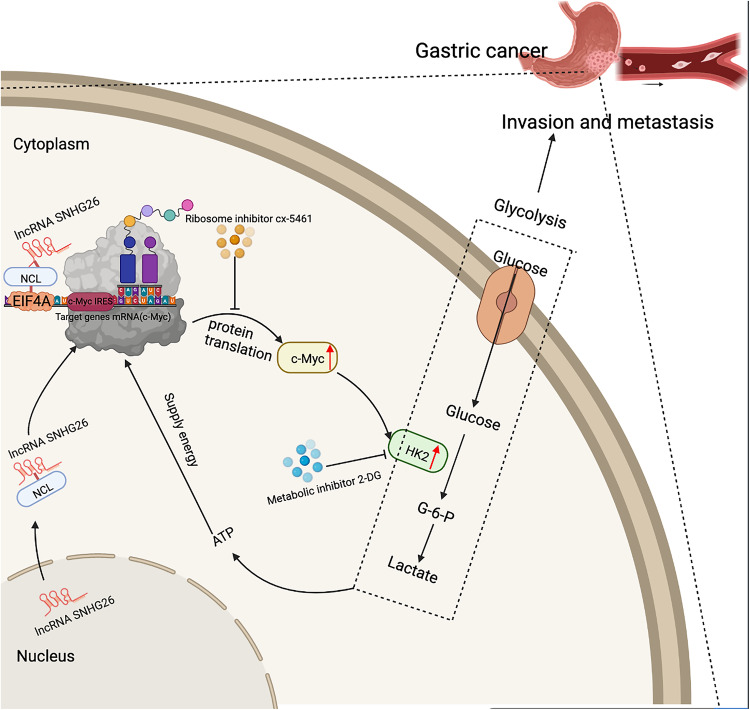
Schematic diagram of the molecular mechanism by which the lncRNA SNHG26 promotes GC metastasis. The lncRNA SNHG26 interacts with NCL and modulates c-Myc expression by increasing its translation to promote energy metabolism via HK2, which in turn promotes GC metastasis. The increase in energy metabolism supplies sufficient energy to promote the translation and expression of c-Myc, forming a positive feedback loop that promotes GC cell migration and invasion.

In the present study, we identified a novel lncRNA termed SNHG26 that was upregulated in GC tissues and predicted poor prognosis in GC patients. The subsequent loss- and gain-of-function analyses revealed that SNHG26 promoted the proliferation and metastasis of GC. Mechanistically, SNHG26 could directly interact with NCL, which binds to c-Myc at the mRNA level. Importantly, SNHG26 could promote c-Myc translation in an IRES-dependent manner via NCL. Furthermore, SNHG26 mediated glycolysis and energy metabolism through the c-Myc/HK2 pathway, promoting the proliferation and migration of GC cells. The increased glycolysis metabolism supplies energy to facilitate mRNA translation, forming a positive feedback loop. In addition, metabolic and translation inhibitors blocked the action of this positive feedback loop, thus inhibiting cancer cell proliferation and mobility, and the combination of both inhibitors could increase the antitumor effect (Fig. 9), indicating the potential therapeutic prospects in GC.

Numerous studies have demonstrated that alterations in lncRNA expression can promote tumorigenesis and metastasis in diverse malignant tumors and serve as biomarkers or therapeutic targets [9, 11]. SNHGs are a family of lncRNAs derived from snoRNAs, and SNHG1 [33, 34], SNHG5 [35], and SNHG20 [36, 37] have been reported to participate in tumor prognosis in various cancers. In the current study, according to the RNA-seq results obtained from six paired EGC tissues, we screened and identified a lncRNA termed SNHG26. However, the biological function of SNHG26 in cancer remains largely unknown. Only one study has been conducted by Jiang et al. and found that SNHG26 promoted cisplatin resistance in tongue squamous cell carcinoma through the PGK1/AKT/mTOR pathway [38]. Here, in vitro and in vivo experiments revealed that SNHG26 promoted proliferation and metastasis in GC, and high SNHG26 expression was associated with poor prognosis. To the best of our knowledge, this is the first study to demonstrate the biological function of SNHG26 in GC.

Many studies have demonstrated that lncRNAs can directly interact with DNA/RNA/protein and exert diverse functions. In the present study, combined with RNA pull-down and RIP assays, we found that SNHG26 could interact with NCL, a nucleocytoplasmic RNA-binding protein that plays multiple roles in cancer [39–41]. Several studies have demonstrated that NCL is mainly distributed in the nucleolus and can bind with ribonucleoprotein (RNP) or precursor ribosomal rRNA, thus affecting ribosomal biogenesis and enhancing the translation of target mRNAs [39]. Interestingly, the Co-IP results showed that NCL directly binds with the ribosomal proteins RPS3 and RPL4, suggesting that SNHG26 might impact mRNA translation via NCL. Indeed, our study revealed that SNHG26 could upregulate rRNA and the ribosomal proteins RPS3 and RPL4, providing a material basis for promoting translation. Subsequent OPP and SUnSET assays demonstrated that SNHG26 promoted mRNA translation in GC.

Interestingly, previous studies have shown that NCL could bind to the c-Myc promoter, promoting its expression via transcription [27]. Consistently, our RIP assay demonstrated that NCL interacted with c-Myc mRNA. Thus, we investigated whether SNHG26 transcriptionally modulates c-Myc expression via NCL. We subsequently found that lncRNA SNHG26 could modulate c-Myc expression at the protein level, whereas no difference was detected at the mRNA level. Moreover, no significant difference in the half-life between MKN-28 vector and MKN-28 SNHG26/overexpressing cells was observed, indicating that SNHG26 might regulate c-Myc expression translationally. In accordance with this hypothesis, the luciferase assay and polysome profiling demonstrated that SNHG26 promoted c-Myc translation in an IRES-dependent manner, indicating that SNHG26 could recruit NCL to Myc RNA and promote its translation. It has been reported in the literature that in viruses, NCL can bind to the IRES site of target gene RNA and recruit translation initiation factors to promote IRES-dependent translation of target genes [39]. Our IP experiments revealed that in human GC cells, SNHG26 promoted the binding of NCL to the translation initiation factor eIF4A and ribosomal protein, suggesting that SNHG26 recruits NCL to Myc RNA and promotes its binding to the translation initiation factor, in turn recruiting ribosomes to Myc RNA to initiate the translation process.

c-Myc, one of the most important oncoproteins, is well known to play critical roles in various biological processes, including energy metabolism [32]. Most tumor cells rely on glycolysis for energy production to maintain rapid proliferation even under aerobic conditions, and conventional mitochondrial oxidative phosphorylation is inhibited, leading to increased glucose consumption and lactate accumulation, a phenomenon called the Warburg effect [31, 42, 43]. In this study, we found that SNHG26 promoted glycolysis and ATP energy metabolism through the c-Myc/HK2 pathway in GC. Dysregulated energy metabolism is not only related to abnormal tumor proliferation but is also involved in tumor metastasis. For example, TIMMDC1 regulates abnormal glycolysis through the AKT/GSK3β/β-catenin signaling pathway, thus promoting GC metastasis [44]. However, the specific regulatory network of energy metabolism involved in tumor metastasis requires further exploration and clarification. In our study, the underlying mechanism is likely the SNHG26-mediated increase in c-Myc mRNA translation, which promotes c-Myc expression and thus mediates energy metabolism, promoting GC metastasis. Moreover, the increased glycolysis metabolism would supply energy to facilitate mRNA translation, forming a positive feedback loop. Recent studies have shown that glucose starvation could block the translation of proteins to save energy for cancer survival [43]. Consistently, our further studies revealed that metabolic and translation inhibitors can block the effect of this positive feedback regulatory loop, and the combination of both could increase the antitumor effect, suggesting potential future therapeutic targets.

In summary, we identified a novel lncRNA, SNHG26, which is upregulated in GC. SNHG26 interacts with NCL and promotes GC proliferation and metastasis by increasing c-Myc translation. Our study revealed a novel mechanism for lncRNAs in the association between translation and metastasis, implicating the SNHG26/NCL/c-Myc axis as a potential therapeutic target in GC.

### Supplementary information

Supplementary materials

original western blots

reproducibility checklist

## References

[1] Bray F, Ferlay J, Soerjomataram I, Siegel RL, Torre LA, Jemal A (2018). Global cancer statistics 2018: GLOBOCAN estimates of incidence and mortality worldwide for 36 cancers in 185 countries. CA Cancer J Clin.

[2] Ferlay J, Colombet M, Soerjomataram I, Mathers C, Parkin DM, Pineros M (2019). Estimating the global cancer incidence and mortality in 2018: GLOBOCAN sources and methods. Int J Cancer.

[3] McLean MH, El-Omar EM (2014). Genetics of gastric cancer. Nat Rev Gastroenterol Hepatol..

[4] Smyth EC, Nilsson M, Grabsch HI, van Grieken NC, Lordick F (2020). Gastric cancer. Lancet.

[5] Zhu XD, Huang MZ, Wang YS, Feng WJ, Chen ZY, He YF (2022). XELOX doublet regimen versus EOX triplet regimen as first-line treatment for advanced gastric cancer: an open-labeled, multicenter, randomized, prospective phase III trial (EXELOX). Cancer Commun.

[6] Yuan L, Xu ZY, Ruan SM, Mo S, Qin JJ, Cheng XD (2020). Long non-coding RNAs towards precision medicine in gastric cancer: early diagnosis, treatment, and drug resistance. Mol cancer.

[7] Bhan A, Soleimani M, Mandal SS (2017). Long noncoding RNA and cancer: a new paradigm. Cancer Res.

[8] Iyer MK, Niknafs YS, Malik R, Singhal U, Sahu A, Hosono Y (2015). The landscape of long noncoding RNAs in the human transcriptome. Nat Genet.

[9] Chen L, Dzakah EE, Shan G (2018). Targetable long non-coding RNAs in cancer treatments. Cancer Lett.

[10] Ma H, Hao Y, Dong X, Gong Q, Chen J, Zhang J (2012). Molecular mechanisms and function prediction of long noncoding RNA. Sci Worl J.

[11] Huarte M (2015). The emerging role of lncRNAs in cancer. Nat Med.

[12] Zhen-Hua W, Yi-Wei G, Li-Qin Z, Jie-Yun Z, Zhe G, Wei-Jian G (2020). Silencing of LncRNA C1RL-AS1 suppresses the malignant phenotype in gastric cancer cells via the AKT/beta-catenin/c-Myc pathway. Front Oncol.

[13] Huang G, Xiang Z, Wu H, He Q, Dou R, Lin Z (2022). The lncRNA BDNF-AS/WDR5/FBXW7 axis mediates ferroptosis in gastric cancer peritoneal metastasis by regulating VDAC3 ubiquitination. Int J Biol Sci.

[14] Sun D, Gou H, Wang D, Li C, Li Y, Su H (2022). LncRNA TNFRSF10A-AS1 promotes gastric cancer by directly binding to oncogenic MPZL1 and is associated with patient outcome. Int J Biol Sci.

[15] Reticker-Flynn NE, Zhang W, Belk JA, Basto PA, Escalante NK, Pilarowski GOW (2022). Lymph node colonization induces tumor-immune tolerance to promote distant metastasis. Cell.

[16] Dragomir MP, Kopetz S, Ajani JA, Calin GA (2020). Non-coding RNAs in GI cancers: from cancer hallmarks to clinical utility. Gut.

[17] Dobin A, Davis CA, Schlesinger F, Drenkow J, Zaleski C, Jha S (2013). STAR: ultrafast universal RNA-seq aligner. Bioinformatics.

[18] Li H, Handsaker B, Wysoker A, Fennell T, Ruan J, Homer N (2009). The sequence alignment/Map format and SAMtools. Bioinformatics.

[19] Anders S, Pyl PT, Huber W (2015). HTSeq–a python framework to work with high-throughput sequencing data. Bioinformatics.

[20] Love MI, Huber W, Anders S (2014). Moderated estimation of fold change and dispersion for RNA-seq data with DESeq2. Genome Biol.

[21] Ritchie ME, Phipson B, Wu D, Hu Y, Law CW, Shi W (2015). limma powers differential expression analyses for RNA-sequencing and microarray studies. Nucleic Acids Res.

[22] Wu ZH, Liu CC, Zhou YQ, Hu LN, Guo WJ (2019). OnclncRNA-626 promotes malignancy of gastric cancer via inactivated the p53 pathway through interacting with SRSF1. Am J cancer Res.

[23] Schmidt EK, Clavarino G, Ceppi M, Pierre P (2009). SUnSET, a nonradioactive method to monitor protein synthesis. Nat methods.

[24] Jia W, Yao Z, Zhao J, Guan Q, Gao L (2017). New perspectives of physiological and pathological functions of nucleolin (NCL). Life Sci.

[25] Wang X, Yu H, Sun W, Kong J, Zhang L, Tang J (2018). The long non-coding RNA CYTOR drives colorectal cancer progression by interacting with NCL and Sam68. Mol cancer.

[26] Okur MN, Lee JH, Osmani W, Kimura R, Demarest TG, Croteau DL (2020). Cockayne syndrome group A and B proteins function in rRNA transcription through nucleolin regulation. Nucleic acids Res.

[27] Gonzalez V, Guo K, Hurley L, Sun D (2009). Identification and characterization of nucleolin as a c-myc G-quadruplex-binding protein. J Biol Chem.

[28] Wu R, Li L, Bai Y, Yu B, Xie C, Wu H (2020). The long noncoding RNA LUCAT1 promotes colorectal cancer cell proliferation by antagonizing nucleolin to regulate MYC expression. Cell death Dis.

[29] Choi SH, Martinez TF, Kim S, Donaldson C, Shokhirev MN, Saghatelian A (2019). CDK12 phosphorylates 4E-BP1 to enable mTORC1-dependent translation and mitotic genome stability. Genes &. development.

[30] Lee HC, Wang H, Baladandayuthapani V, Lin H, He J, Jones RJ (2017). RNA polymerase I inhibition with CX-5461 as a novel therapeutic strategy to target MYC in multiple myeloma. Br J Haematol.

[31] Cantor JR, Sabatini DM (2012). Cancer cell metabolism: one hallmark, many faces. Cancer Discov.

[32] Hung CL, Wang LY, Yu YL, Chen HW, Srivastava S, Petrovics G (2014). A long noncoding RNA connects c-Myc to tumor metabolism. Proc Natl Acad Sci USA.

[33] Sun Y, Wei G, Luo H, Wu W, Skogerbo G, Luo J (2017). The long noncoding RNA SNHG1 promotes tumor growth through regulating transcription of both local and distal genes. Oncogene.

[34] Lu Q, Shan S, Li Y, Zhu D, Jin W, Ren T (2018). Long noncoding RNA SNHG1 promotes non-small cell lung cancer progression by up-regulating MTDH via sponging miR-145-5p. FASEB J : Off Publ Fed Am Soc Exp Biol.

[35] Damas ND, Marcatti M, Come C, Christensen LL, Nielsen MM, Baumgartner R (2016). SNHG5 promotes colorectal cancer cell survival by counteracting STAU1-mediated mRNA destabilization. Nat Commun.

[36] Chen Z, Chen X, Chen P, Yu S, Nie F, Lu B (2017). Long non-coding RNA SNHG20 promotes non-small cell lung cancer cell proliferation and migration by epigenetically silencing of P21 expression. Cell death Dis.

[37] Wang Y, Fu J, Yang L, Liang Z (2021). Long noncoding RNA SNHG20 promotes colorectal cancer cell proliferation, migration and invasion via miR495/STAT3 axis. Mol Med Rep..

[38] Jiang Q, Wang Z, Qi Q, Li J, Xin Y, Qiu J (2022). lncRNA SNHG26 promoted the growth, metastasis, and cisplatin resistance of tongue squamous cell carcinoma through PGK1/Akt/mTOR signal pathway. Mol Ther oncolytics.

[39] Abdelmohsen K, Tominaga K, Lee EK, Srikantan S, Kang MJ, Kim MM (2011). Enhanced translation by nucleolin via G-rich elements in coding and non-coding regions of target mRNAs. Nucleic acids Res.

[40] Xu C, Wang Y, Tu Q, Zhang Z, Chen M, Mwangi J (2019). Targeting surface nucleolin induces autophagy-dependent cell death in pancreatic cancer via AMPK activation. Oncogene.

[41] Pichiorri F, Palmieri D, De Luca L, Consiglio J, You J, Rocci A (2013). In vivo NCL targeting affects breast cancer aggressiveness through miRNA regulation. J Exp Med.

[42] Liu H, Luo J, Luan S, He C, Li Z (2019). Long non-coding RNAs involved in cancer metabolic reprogramming. Cell Mol Life Sci: CMLS.

[43] Guo T, Bai YH, Cheng XJ, Han HB, Du H, Hu Y (2021). Insulin gene enhancer protein 1 mediates glycolysis and tumorigenesis of gastric cancer through regulating glucose transporter 4. Cancer Commun.

[44] Liu Y, Huang Y, Zhang J, Pei C, Hu J, Lyu J (2018). TIMMDC1 knockdown inhibits growth and metastasis of gastric cancer cells through metabolic inhibition and AKT/GSK3beta/beta-catenin signaling pathway. Int J Biol Sci.

