# Supplementary material for: LncRNA SNHG26 promotes gastric cancer progression and metastasis by inducing c-Myc protein translation and an energy metabolism positive feedback loop
Source: Cell Death Dis. 2024 Mar 29;15(3):236. doi: 10.1038/s41419-024-06607-8 (PMC10980773; doi:10.1038/s41419-024-06607-8)
Supplement: Supplementary file 1 — Supplementary materials [file 41419_2024_6607_MOESM1_ESM.pdf]

Supplementary materials

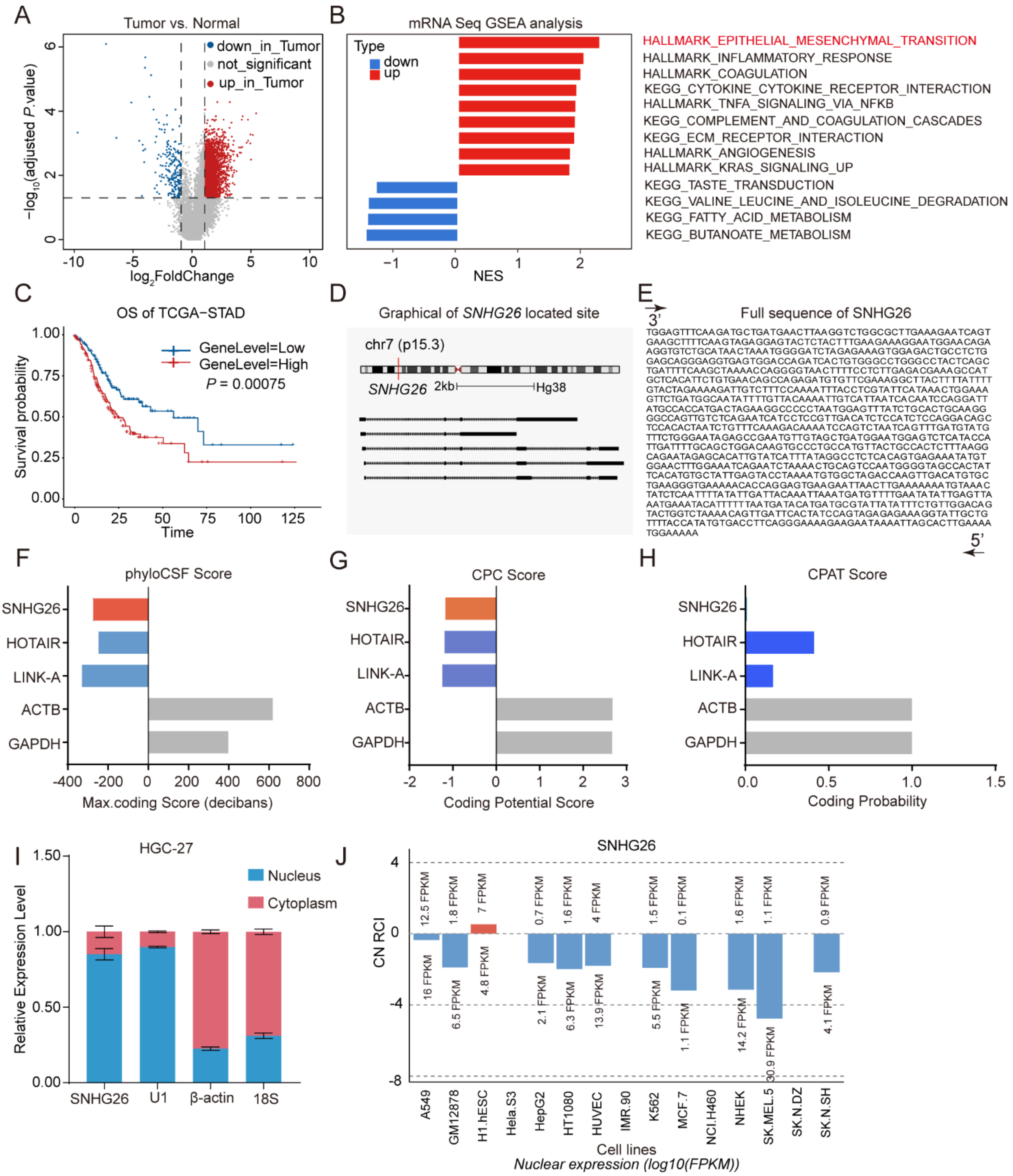

Supplementary Figure 1. Identification and characterization of lncRNA SNHG26 in GC.

A. Volcano map of mRNA expression in RNA-seq. **B.** GSEA of mRNA expression in RNA-seq. **C.** Overall survival analysis of SNHG26 in TCGA-STAD. **D.** Schematic representation of the genomic locus and isoforms of SNHG26

in the UCSC Genome browser (http://genome.ucsc.edu/). **E.** Sequence of the major transcript of SNHG26 from RACE. **F-H.** The coding potential of SNHG26 was analyzed using PhyloCSF codon substitution frequency analysis (F), the Coding Potential Calculator (CPC, G), and the Coding Potential Assessment Tool (CPAT, H). HOTARI and LINK-A served as noncoding RNA controls, and ACTB and GAPDH served as coding RNA controls. **I.** The subcellular location of lncRNA SNHG26 in HGC-27 cells, β-actin and 18S served as the cytoplasmic internal control, and U1 served as the nuclear internal control. **J.** The subcellular location of lncRNA SNHG26 in 15 human cancer cell lines (http://lncatlas.crg.eu/).

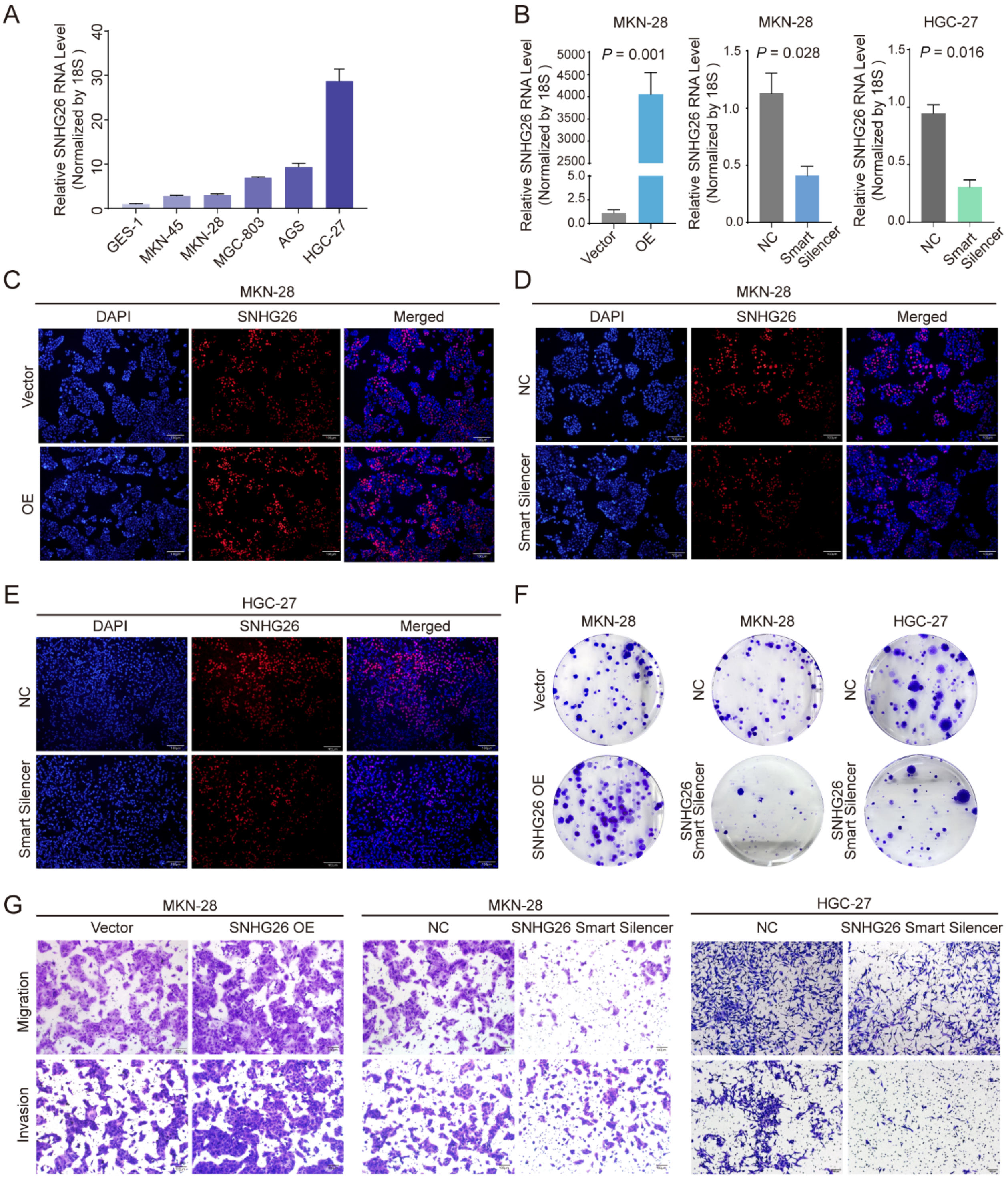

Supplementary Figure 2. SNHG26 promotes proliferation and metastasis *in vitro* in GC.

A. Various levels of SNHG26 expression in GC cell lines. **B.** The overexpression and silencing efficacy of SNHG26 lentivirus and smart silencers in MKN-28 and HGC-27 cells measured by qPCR. **C-E.** Representative images of the EdU assay after ectopic expression of SNHG26. Bars: 100 μM. **F.** Representative images of the

colony formation assay after modulating SNHG26 expression. **G.** Representative images of migration and invasion assays after modulating SNHG26 expression. Bars: 100 μM. All data are from three independent experiments. The data are presented as the mean ± SD values ($n \geq 3$). *$P < 0.05$; **$P < 0.01$; ***$P < 0.001$; ****$P < 0.0001$.

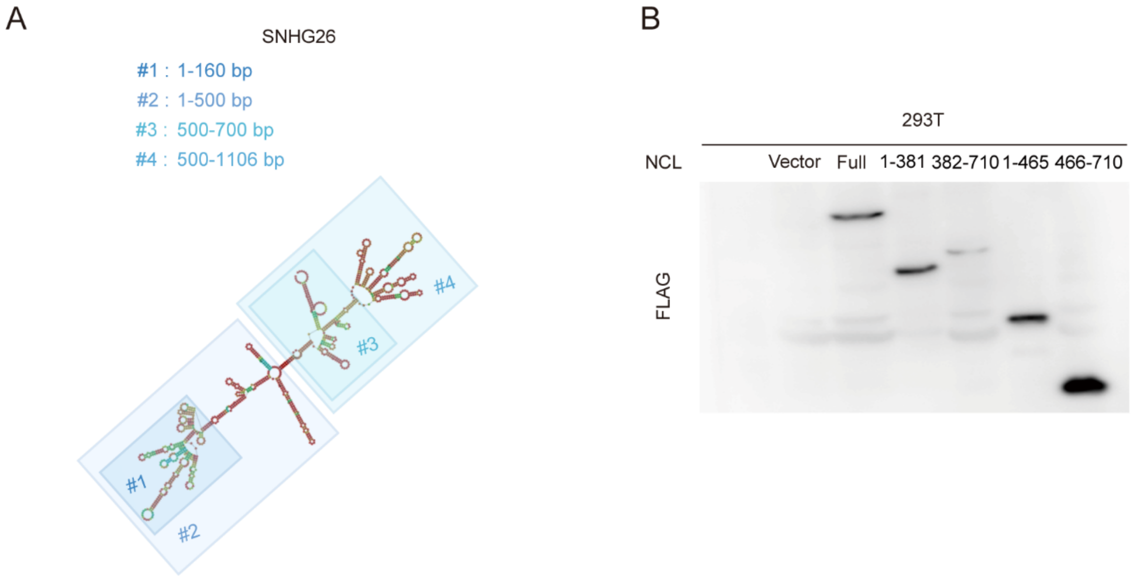

Supplementary Figure 3. Identification of SNHG26-associated proteins.

A. Graphic illustration of the predicted SNHG26 secondary structure analyzed by LNCipedia (http://www.lncipedia.org) and the truncated fragments of SNHG26 according to the stem-loop structure. **B.** Deletion mapping to identify the domains of NCL that bind to SNHG26.

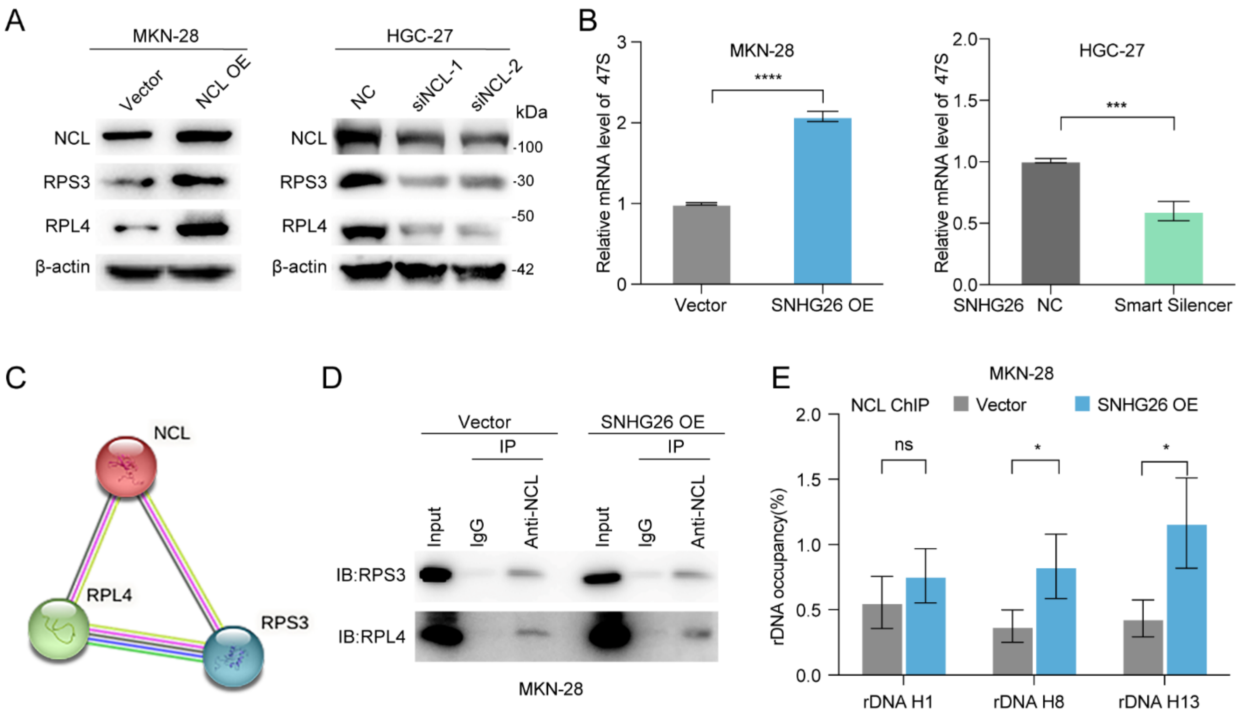

Supplementary Figure 4. SNHG26 promotes pre-rRNA synthesis and facilitates NCL-regulated rDNA transcription.

A. MKN-28 cells were infected with NCL overexpression plasmid (NCL-OE) or control empty vector. HGC-27 cells were transfected with NCL small interfering RNA (siNCL) or NC siRNAs (NC). The expression of NCL, RPS3, and RPL4 was examined by western blotting. **B.** Expression of 47S rRNA was measured by qPCR after overexpression or knockdown of SNHG26 in MKN-28 and HGC-27 cells. **C.** The STRING website predicted that NCL binds to RPS3 and RPL4. **D.** A Co-IP assay verified the presence of interactions between NCL and RPS3 and RPL4 in SNHG26 vector and overexpressed MKN-28 cells. **E.** ChIP analysis was performed using the antibody to NCL on extracts of SNHG26 overexpressed cells or vector cells. All data are from three independent experiments. The data are presented as the mean ± SD values ($n \geq 3$). *$P < 0.05$; **$P < 0.01$; ***$P < 0.001$; ****$P < 0.0001$.

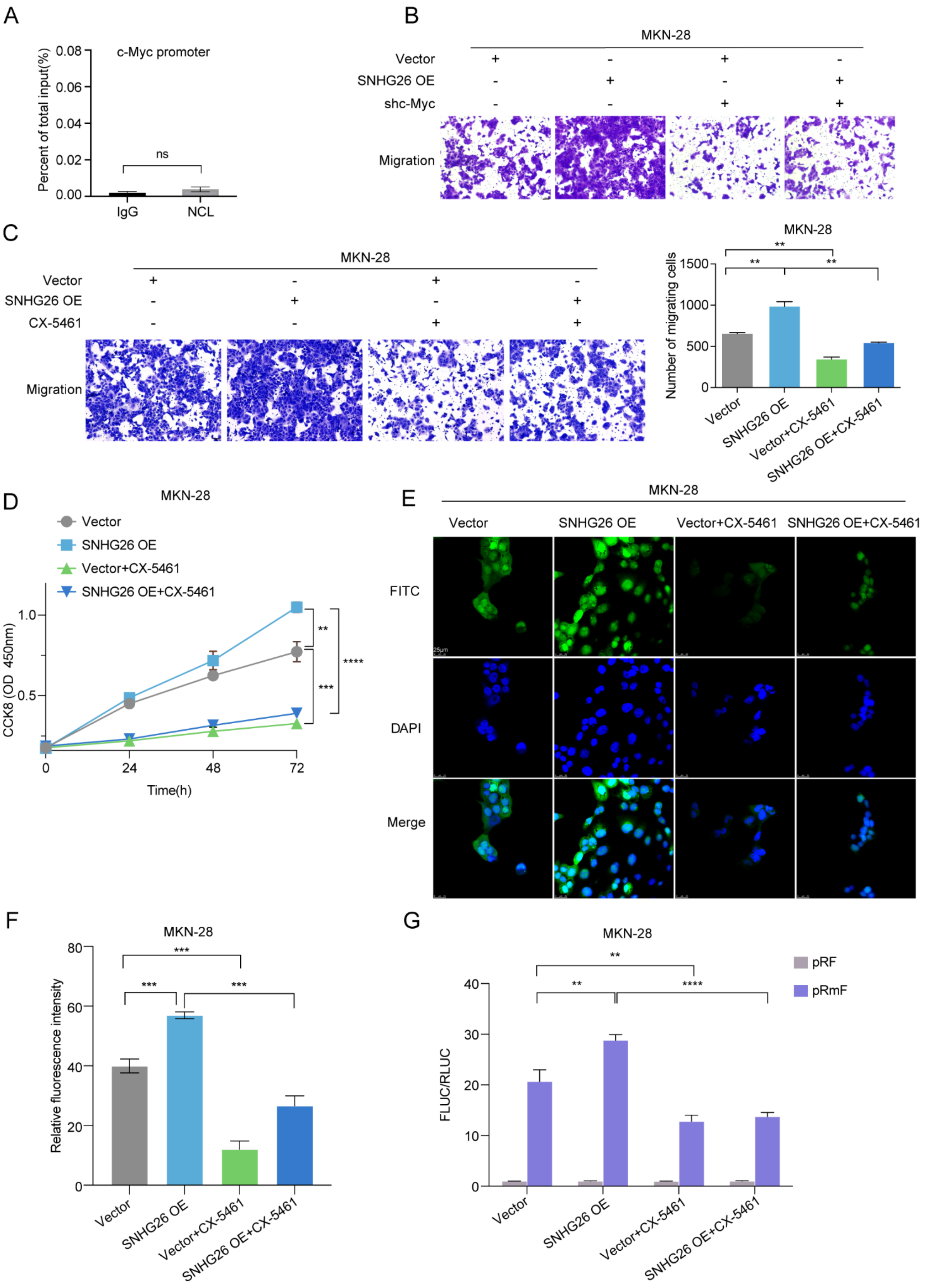

Supplementary Figure 5. LncRNA SNHG26 promotes the proliferation and migration of GC cells by

stimulating c-Myc translation in an NCL-dependent manner.

A. A ChIP analysis was performed using an antibody to NCL on extracts of MKN-28 cells. **B.** Representative images of the Transwell assay after c-Myc knockdown in SNHG26 overexpressed MNK-28 cells. Scale bar = 100 μM. **C-D.** CCK-8 and Transwell assays showed that the increased proliferation and migration of SNHG26-overexpressing cells was significantly reversed by adding CX-5461 (500 nM). Scale bars = 100 μM. **E.** Protein synthesis was measured after adding the ribosome inhibitor CX-5461 to SNHG26 overexpressed MNK-28 cells (OPP, green; DAPI, blue). Scale bar = 25 μM. **F.** Quantification of Alexa 488 fluorescence intensity. **G.** The expression of firefly with Renilla luciferase was assessed after adding CX-5461 to SNHG26 overexpressed MNK-28 cells. All data are from three independent experiments. The data are presented as the mean ± SD values ($n \geq 3$). $^{*}P < 0.05$; $^{**}P < 0.01$; $^{***}P < 0.001$; $^{****}P < 0.0001$.

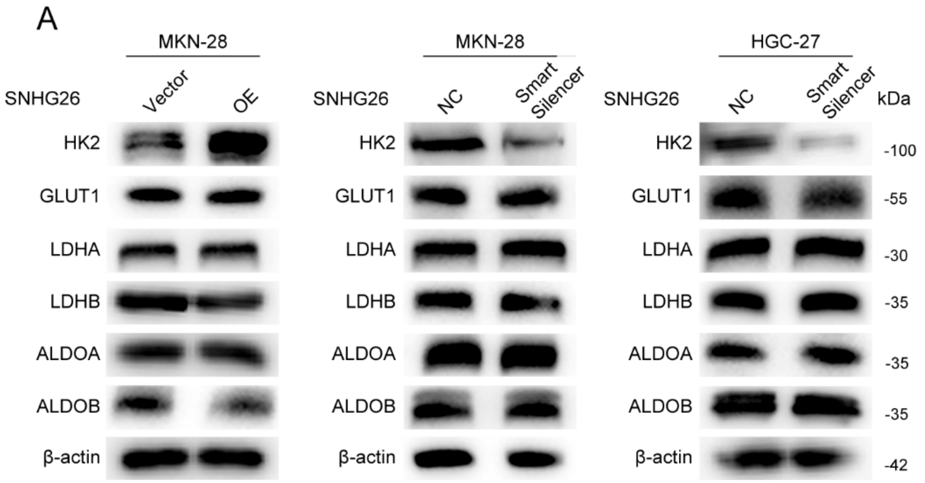

Supplementary Figure 6. SNHG26 regulates energy metabolism through the c-Myc/HK2 pathway.

A. Metabolism-associated protein expressions were detected by western blotting in MKN-28 and HGC-27 cells after modulating SNHG26 status.

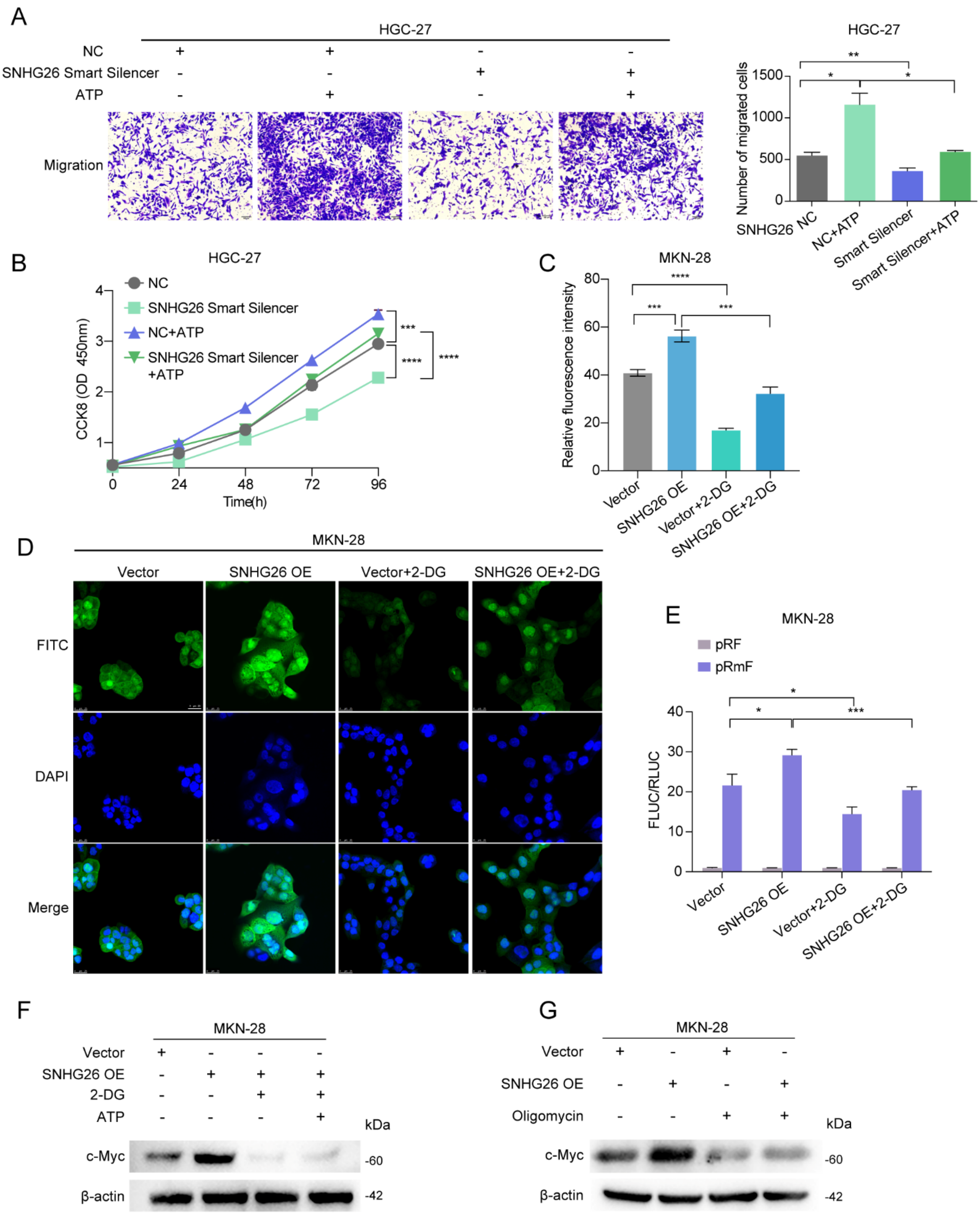

Supplementary Figure 7. SNHG26 promotes energy production by regulating the c-Myc/HK2 pathway, and positive feedback promotes the translation and expression of c-Myc.

A-B. Transwell and CCK-8 assays showed that the increased migration and proliferation of SNHG26-knockdown

cells were significantly rescued by adding ATP (100 nM). Scale bar = 100 μM. **C.** Quantification of protein synthesis levels in SNHG26-overexpressed MKN-28 cells after the addition of 2-DG. **D.** Protein synthesis was measured after the addition of the metabolic inhibitor 2-DG in MKN-28 cells overexpressing SNHG26 (OPP, green; DAPI, blue). Scale bar = 25 μM. **E.** The expression of firefly with Renilla luciferase was assessed after adding 2-DG in SNHG26-overexpressed MKN-28 cells. **F.** Western blotting showing that the increase in c-Myc levels was significantly reversed when ATP was added to 2-DG-treated MKN-28 cells. **G.** Western blotting showing that the elevation of c-Myc levels was significantly reversed in MKN-28 cells treated with oligomycin (10 μg/ml) for 1.5 h. All data are from three independent experiments. The data are presented as the mean ± SD values ($n \geq 3$). *$P < 0.05$; **$P < 0.01$; ***$P < 0.001$; ****$P < 0.0001$.

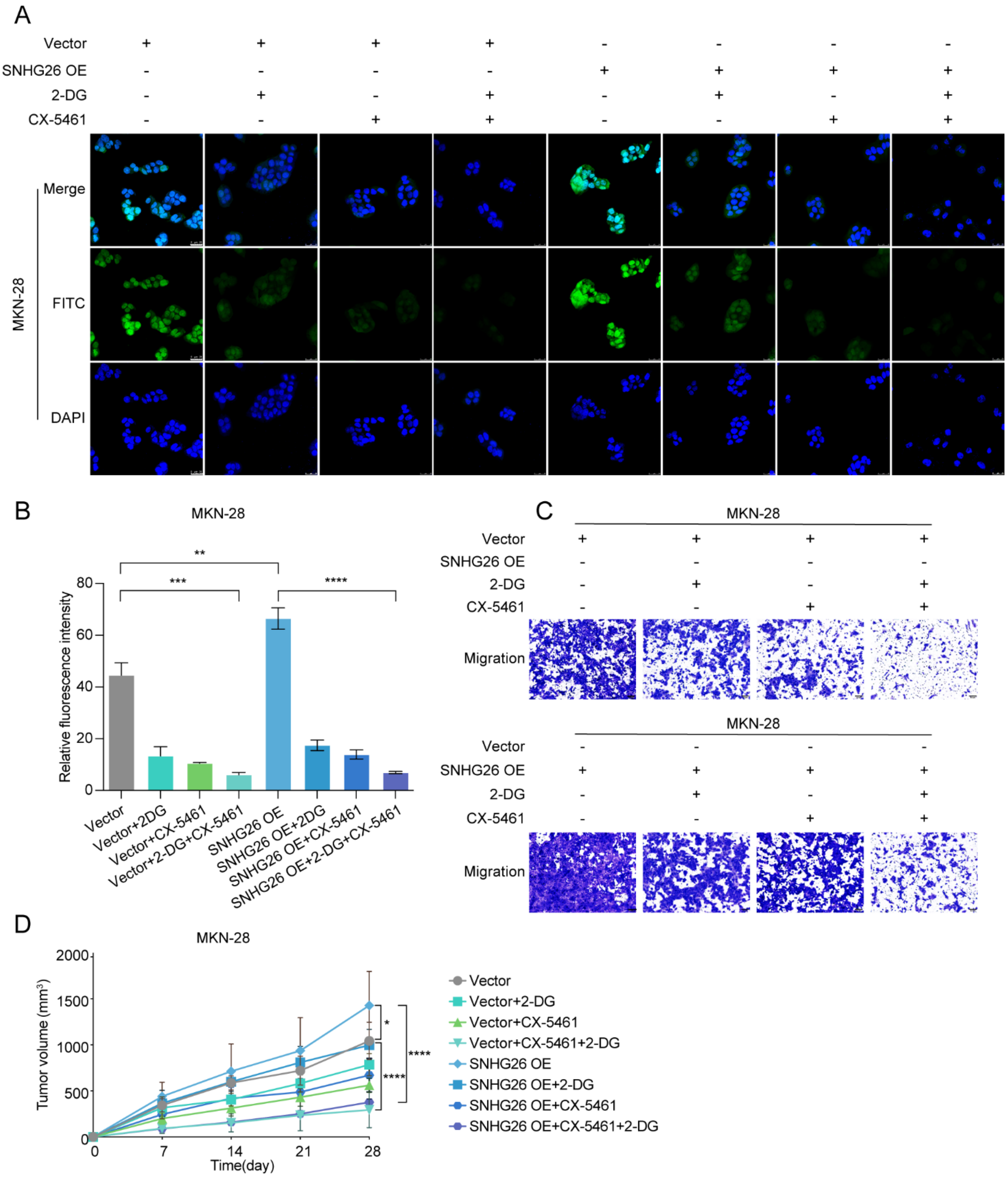

Supplementary Figure 8. The combination of a translation inhibitor (CX-5461) and a metabolic inhibitor (2-DG) effectively inhibits protein synthesis and migration in GC cells.

A. Representative fluorescence micrographs of control or SNHG26-overexpressing MKN-28 cells treated with the metabolic inhibitor 2-DG alone, ribosome inhibitor CX-5461 alone, and the combination of both drugs after the

addition of OPP-labeled nascent protein (green) and nuclear staining (DAPI; blue) using the Click-iT assay. Scale bar = 25 μM. **B.** Quantification of Alexa 488 fluorescence intensity. **C.** Representative images of the Transwell assay after treatment with 2-DG alone, CX-5461 alone, and a combination of 2-DG and CX-5461. Scale bar = 100 μM. **D.** Growth curves of xenograft tumors in nude mice treated as described above. All data are from three independent experiments. The data are presented as the mean ± SD values ($n \geq 3$). *$P < 0.05$; **$P < 0.01$; ***$P < 0.001$; ****$P < 0.0001$.

Supplementary Table 1. PCR primer sequences

Primer name	primer sequence (5'-3')
GAPDH-Forward	ACCCAGAAGACTGTGGATGG
GAPDH-Reverse	TTCTAGACGGCAGGTCAGGT
c-Myc-Forward	GGCTCCTGGCAAAAGGTCA
c-Myc-Reverse	CTGCGTAGTTGTGCTGATGT
HK2-Forward	CGACAGCATCATTGTTAAGGAG
HK2-Reverse	GCAGGAAAGACACATCACATTT
lncRNA SNHG26-Forward	GGCGCTTGAAAGAATCAGTGAAG
lncRNA SNHG26-Reverse	CCACAGGATCTGGTCCACTC
β-actin-Forward	TTGGCCAGGGGTGCTAAG
β-actin-Reverse	AGCCAAAAGGGTCATCATCTC
U1-Forward	GAAACTCGACTGCATAATTTGIGGTAG
U1-Reverse	CTTGGCGTACAGTCTGTTTTTGAAACTC
18S-Forward	CGGQTACCACATCCAAGGAA
18S -Reverse	GCTGGAATTACCGCGGCT
NCL-Forward	GGTGGTCGTTTCCCCAACAAA
NCL-Reverse	GCCAGGTGTGGTAACTGCT
47S-Forward	TGTCAGGCGTTCTCGTCTC
47S -Reverse	GAGAGCACGACGTCACCAC
SNHG26-pulldown-sense-Forward	GATCACTAATACGACTCACTATAGGTG GAGTTTCAAGATGCTGAT
SNHG26-pulldown-sense-Reverse	TTTTTCCATTTTCAAGTGCT
SNHG26-pulldown-antisense-Forward	TGGAGTTTCAAGATGCTGAT
SNHG26-pulldown-antisense-Reverse	GATCACTAATACGACTCACTATAGGTTT TTCCATTTTCAAGTGCT
SNHG26-RACE-Forward	TTAAGGTCTGGCGCTTG
SNHG26-RACE-Reverse	CCATTTTCAAGTGCTAATT
c-MYC ChIP Forward	AGTCTTTGATATGGAGACAGACTAG
c-MYC ChIP Reverse	GAGCCACCATACCAAGCCTGTTTGC
rDNA promoter H1 Forward	GGCGGTTTGAGTGAGACGAGA
rDNA promoter H1 Reverse	ACGTGCGCTCACCGAGAGCAG
rDNA promoter H8 Forward	AGTCGGGTTGCTTGGGAATGC
rDNA promoter H8 Reverse	CCCTTACGGTACTTGTTGACT
rDNA promoter H13 Forward	ACCTGGCGCTAAACCATTCGT
rDNA promoter H13 Reverse	GGACAAACCCTTGTGTCGAGG

Supplementary Table 2. Antibodies

Antibody name	Companies	Dilution ratio
NCL	Proteintech	1:1000
c-Myc	Proteintech	1:1000
RPS3	Proteintech	1:1000
RPL4	Proteintech	1:1000
Puromycin	Thermo Fisher Scientific	1:1000
HK2	Proteintech	1:1000
GLUT1	Proteintech	1:1000
LDHA	Proteintech	1:1000
LDHB	Proteintech	1:1000
ALDOA	Proteintech	1:1000
ALDOB	Proteintech	1:1000
E-Cadherin	Cell Signaling Technology	1:1000
Vimentin	Cell Signaling Technology	1:1000
Snail	Cell Signaling Technology	1:1000
N-Cadherin	Proteintech	1:1000
β-actin	Proteintech	1:50000
Goat Anti-Rabbit Mouse IgG-HRP	Absmart	1:5000
DYKDDDDK tag	Proteintech	1:5000
