# Supplementary figures and images for: LncRNA SNHG26 promotes gastric cancer progression and metastasis by inducing c-Myc protein translation and an energy metabolism positive feedback loop
Source: Cell Death Dis. 2024 Mar 29;15(3):236. doi: 10.1038/s41419-024-06607-8 (PMC10980773; doi:10.1038/s41419-024-06607-8)

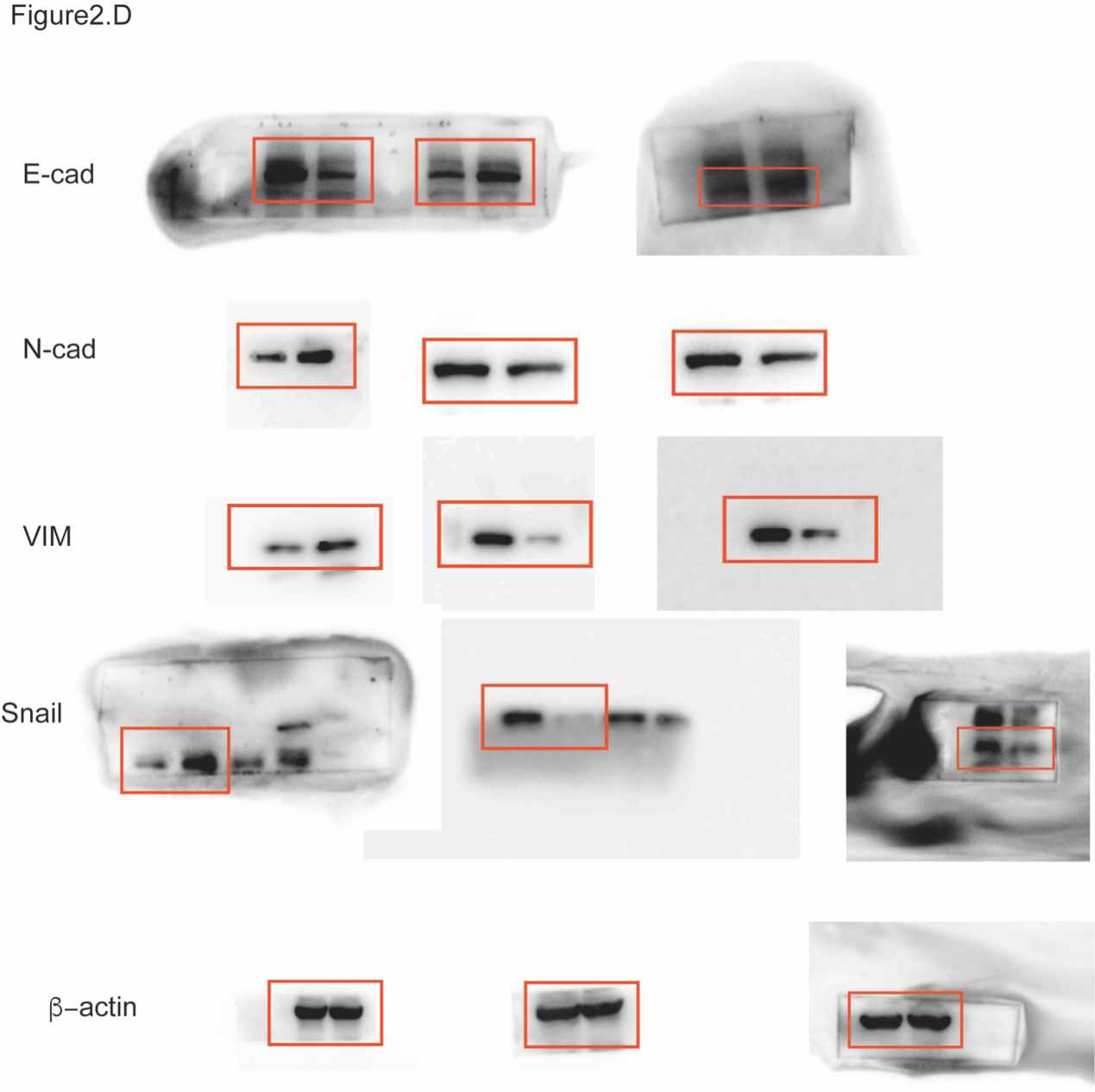


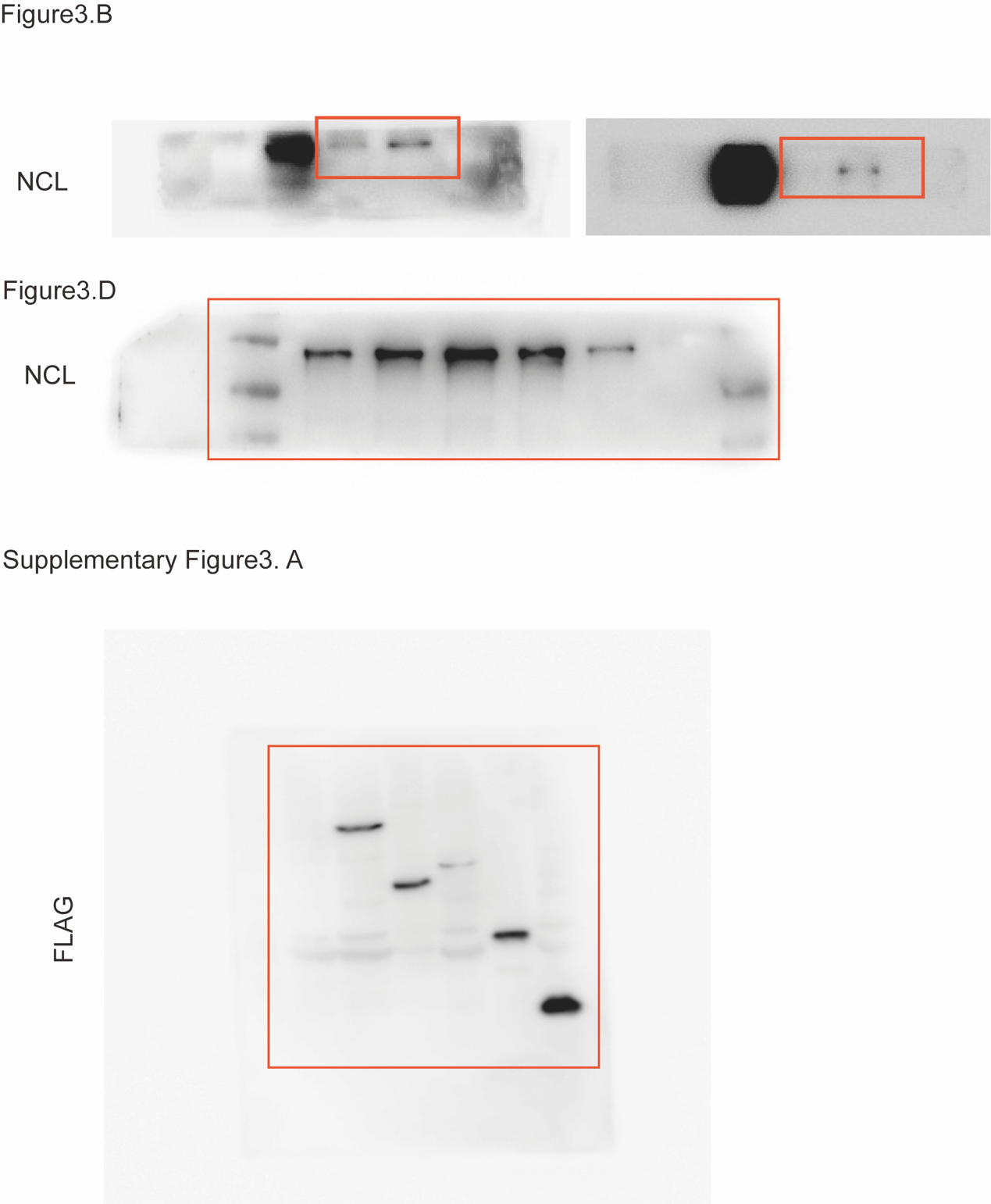


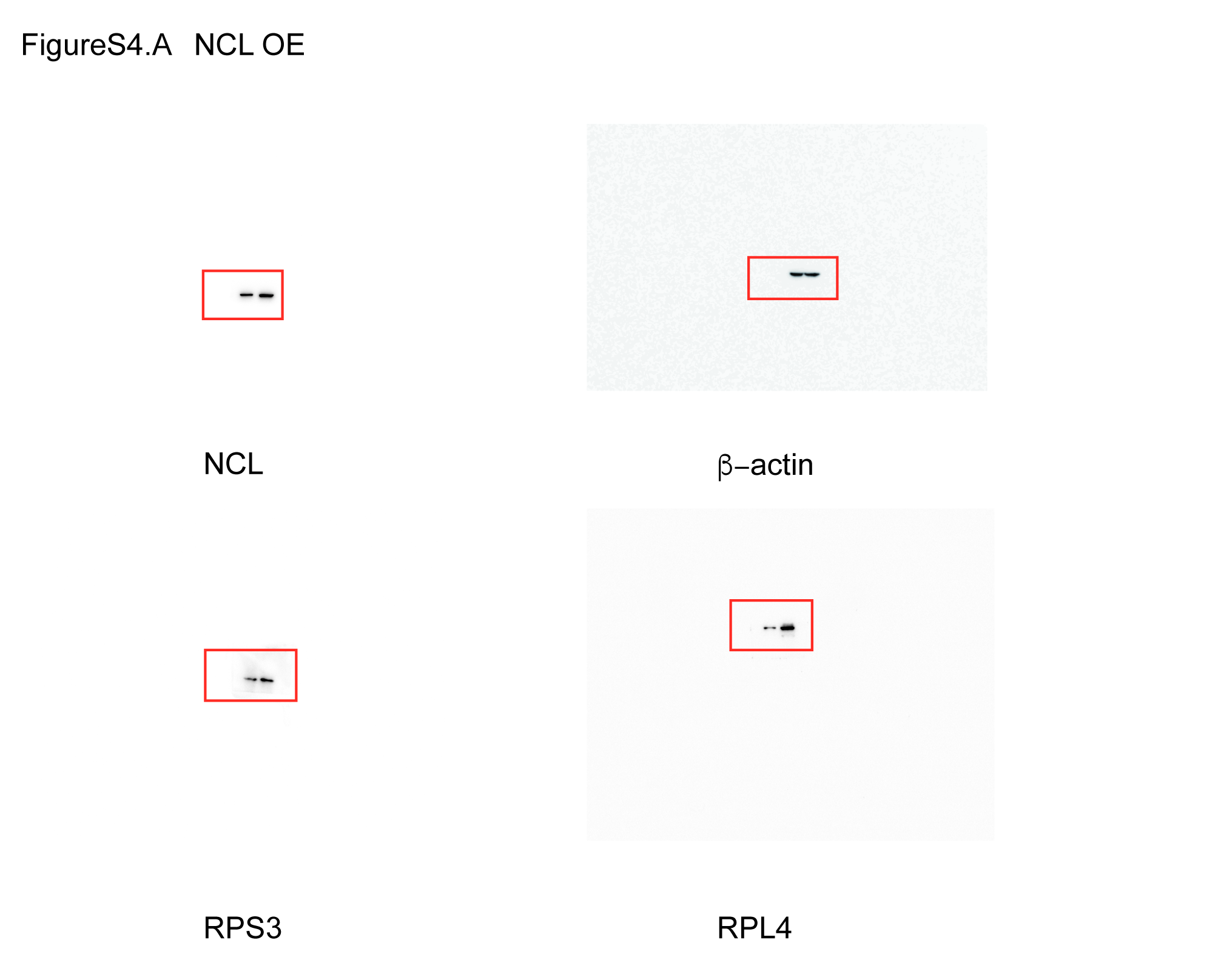

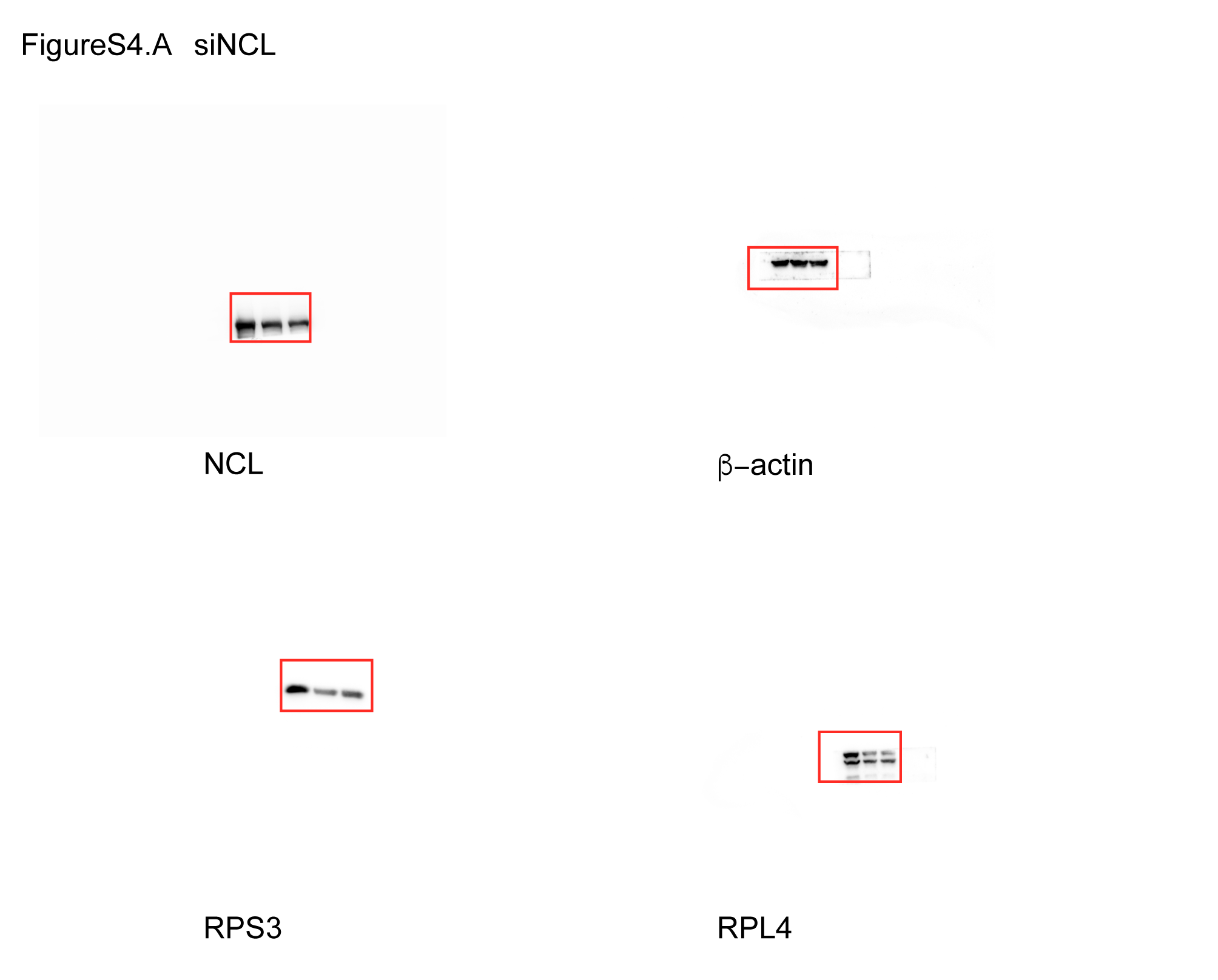

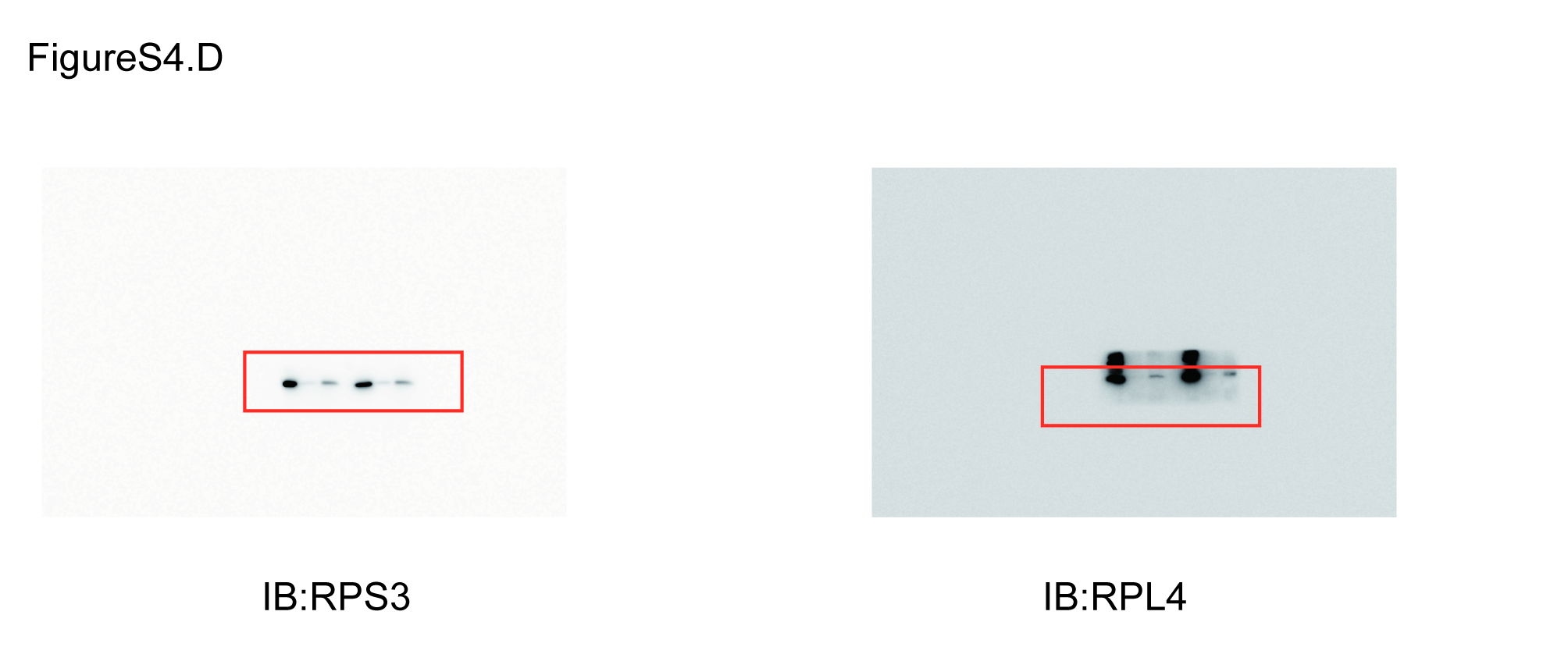

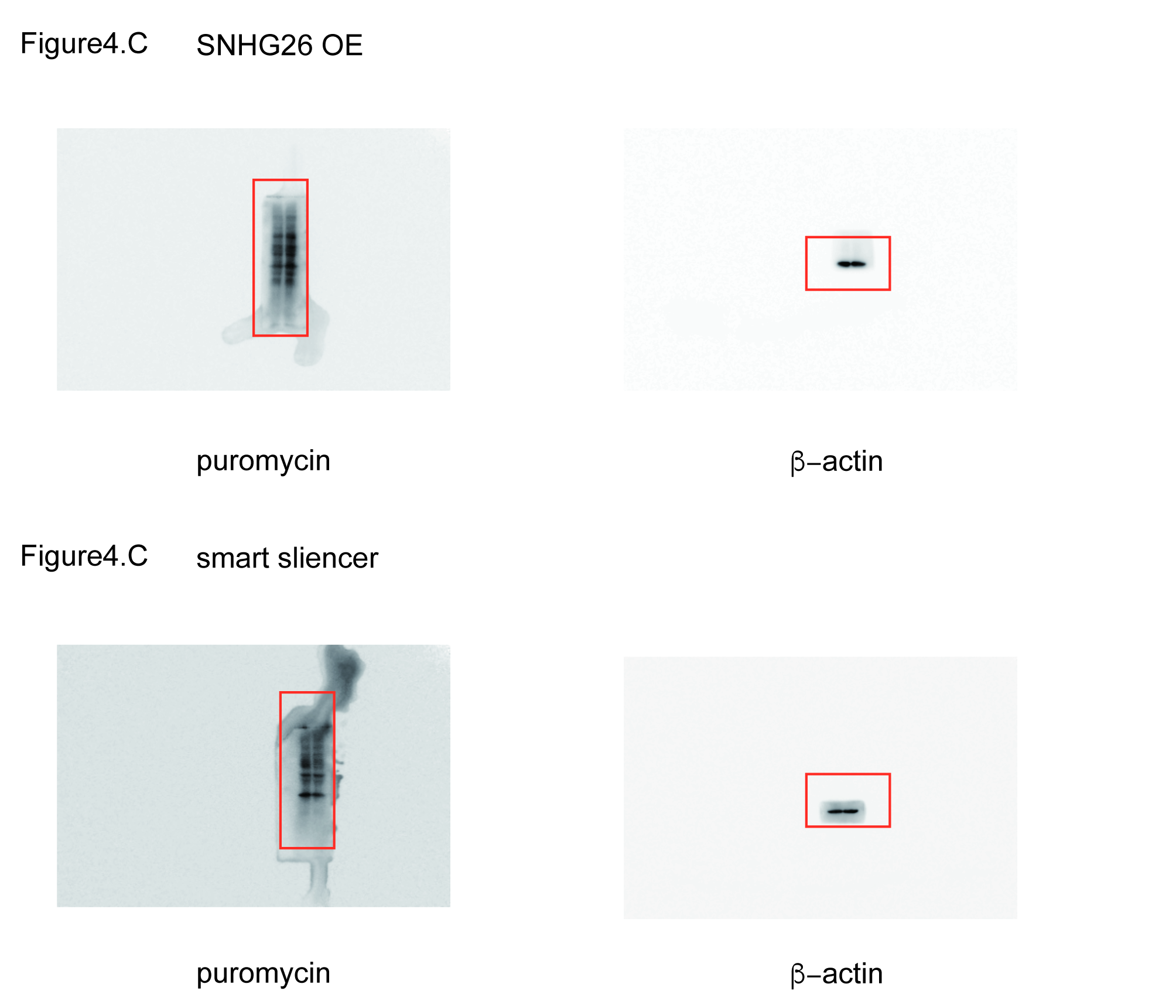

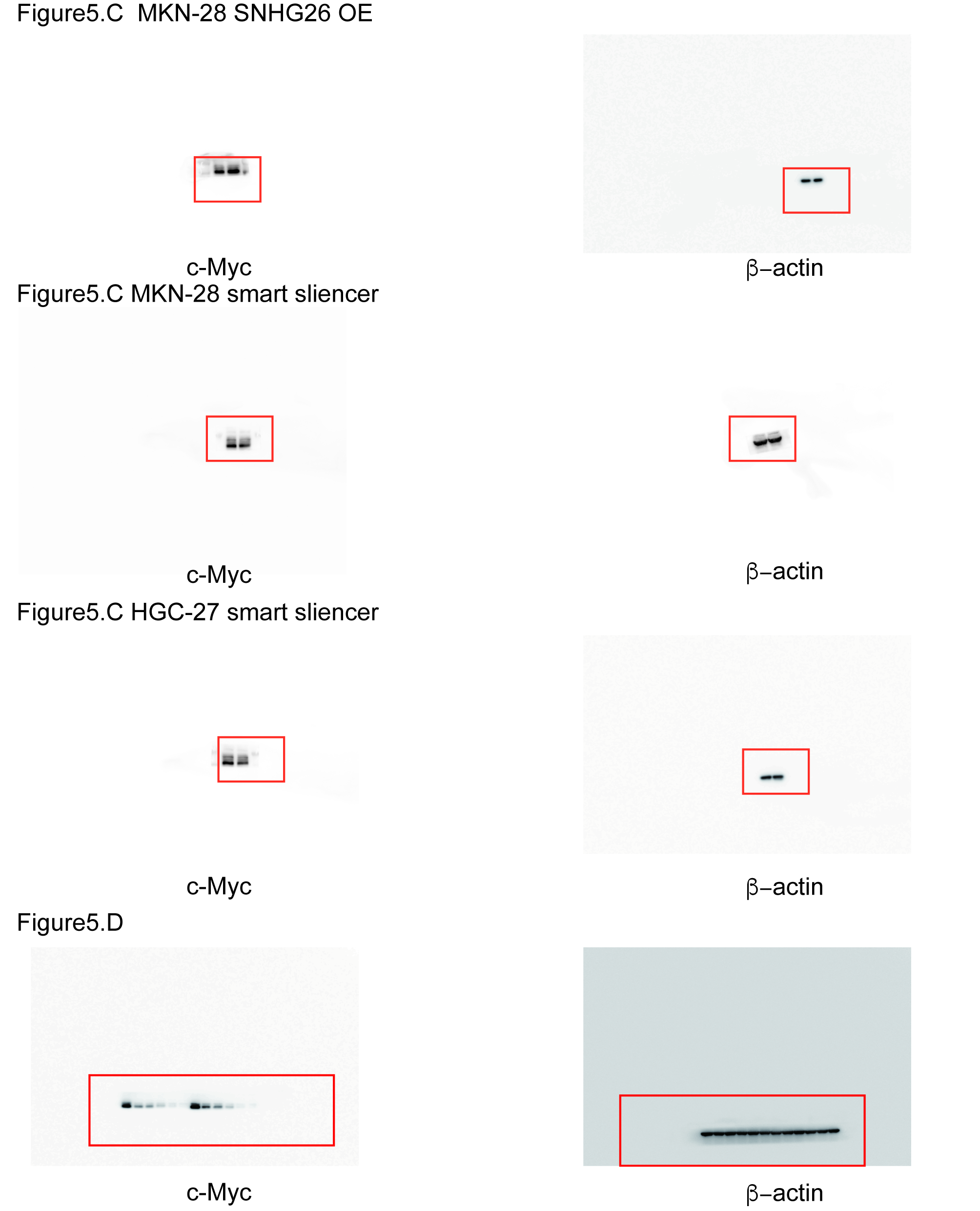

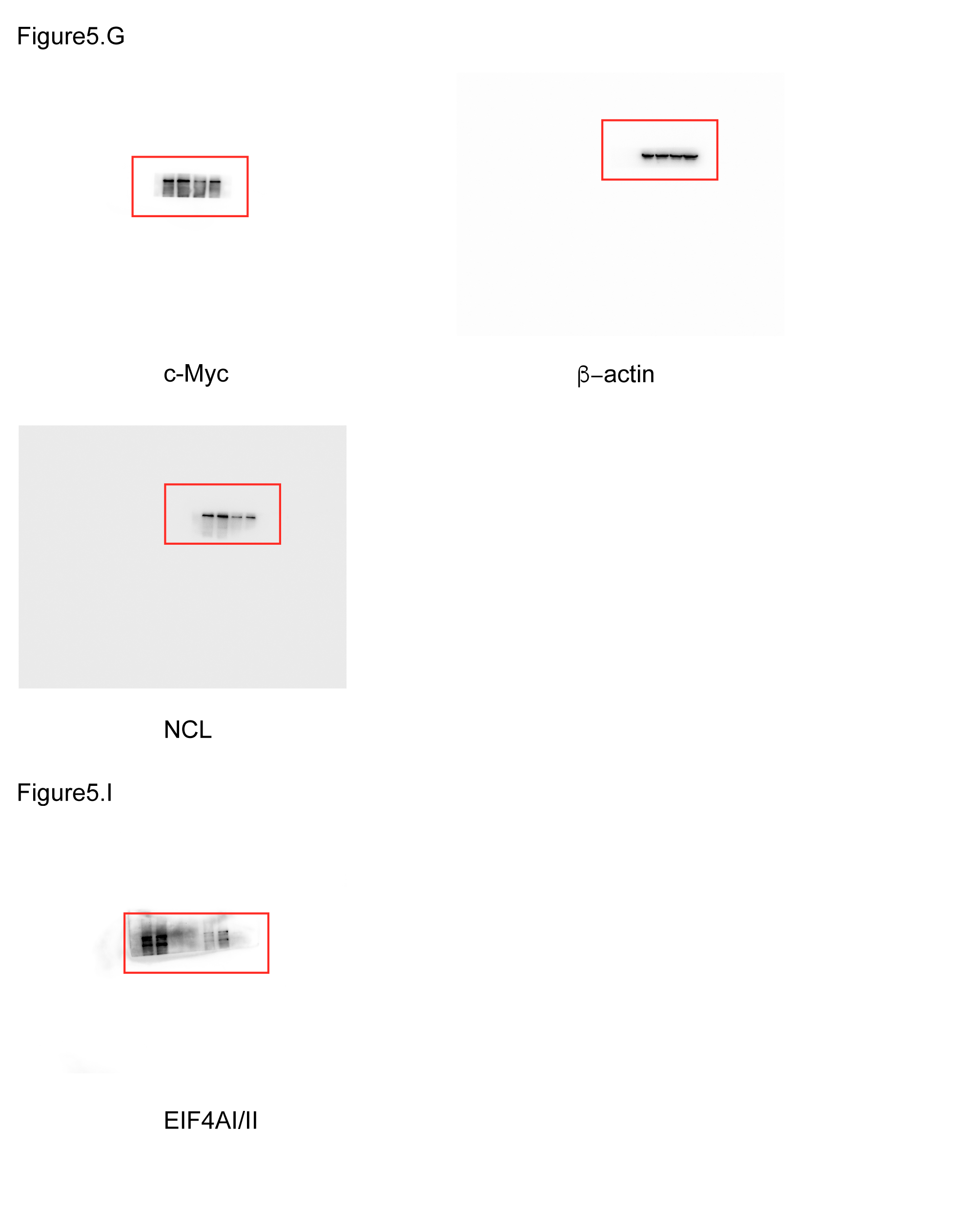

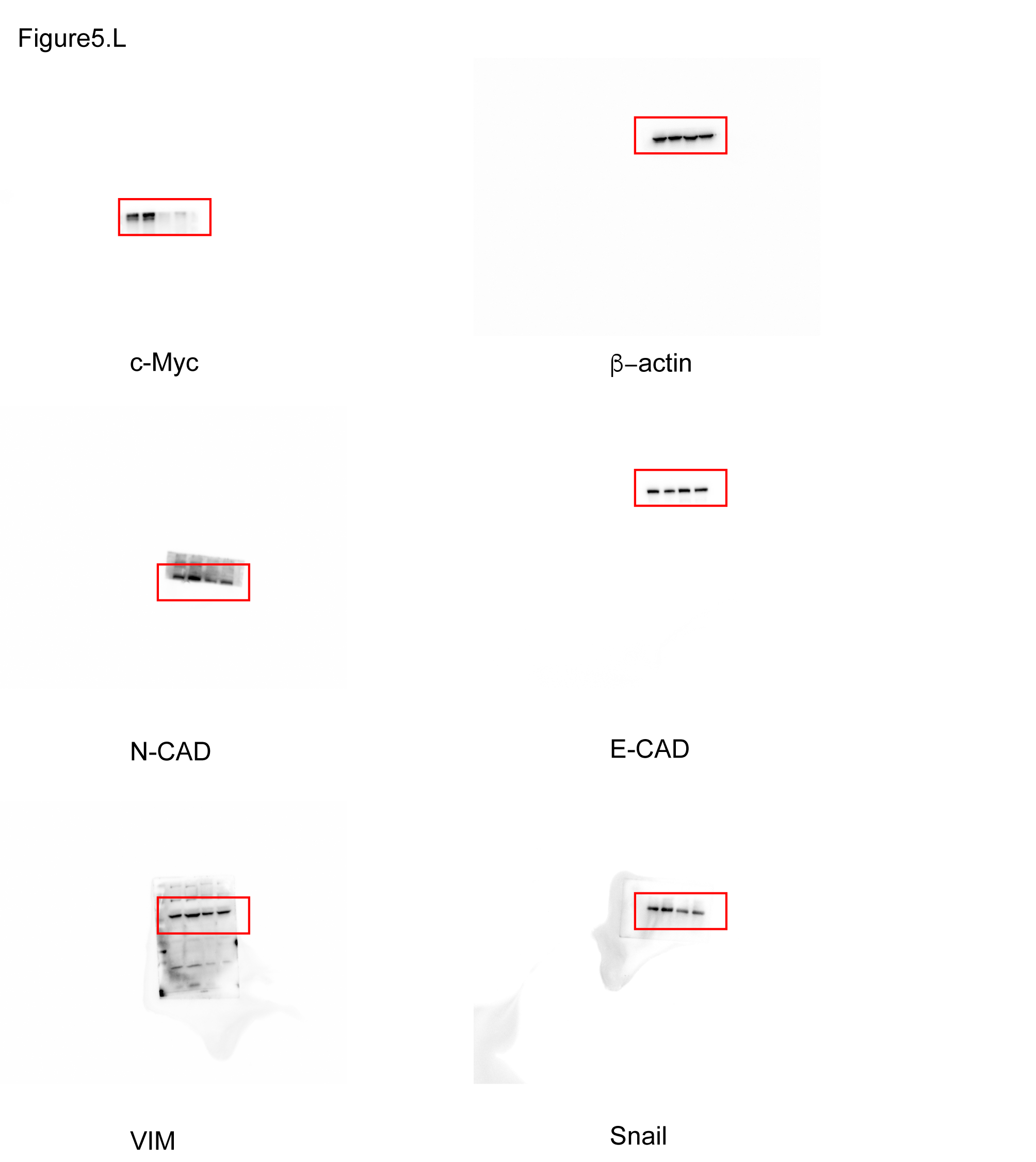

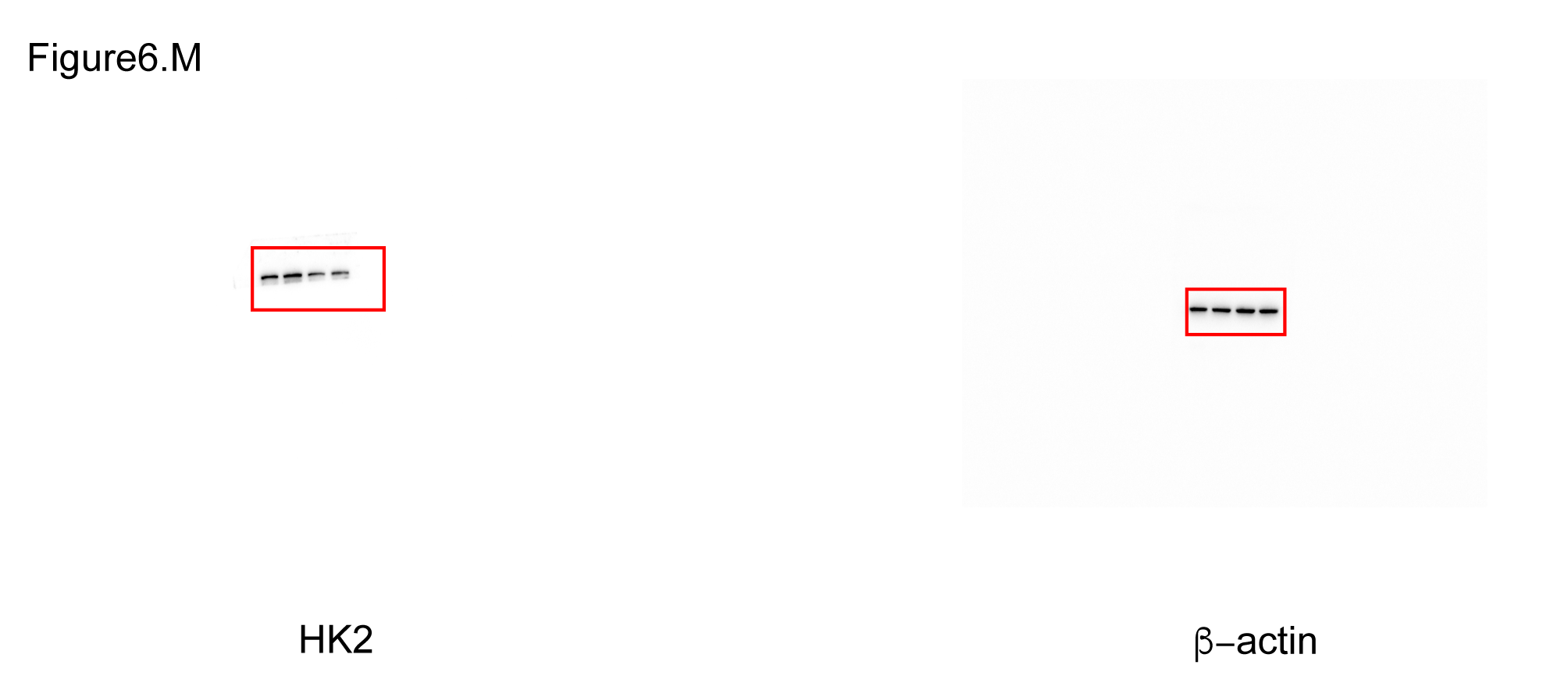

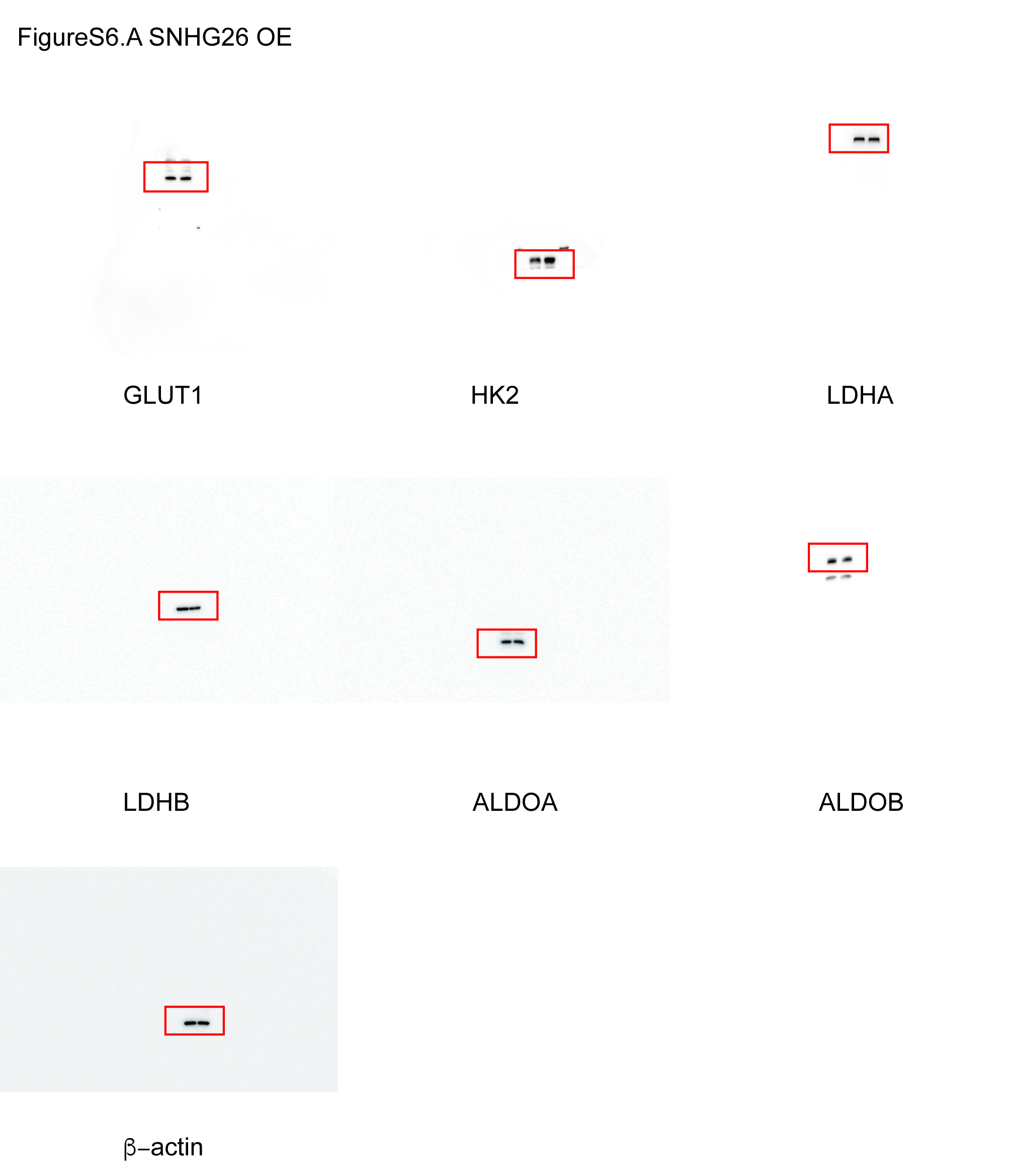

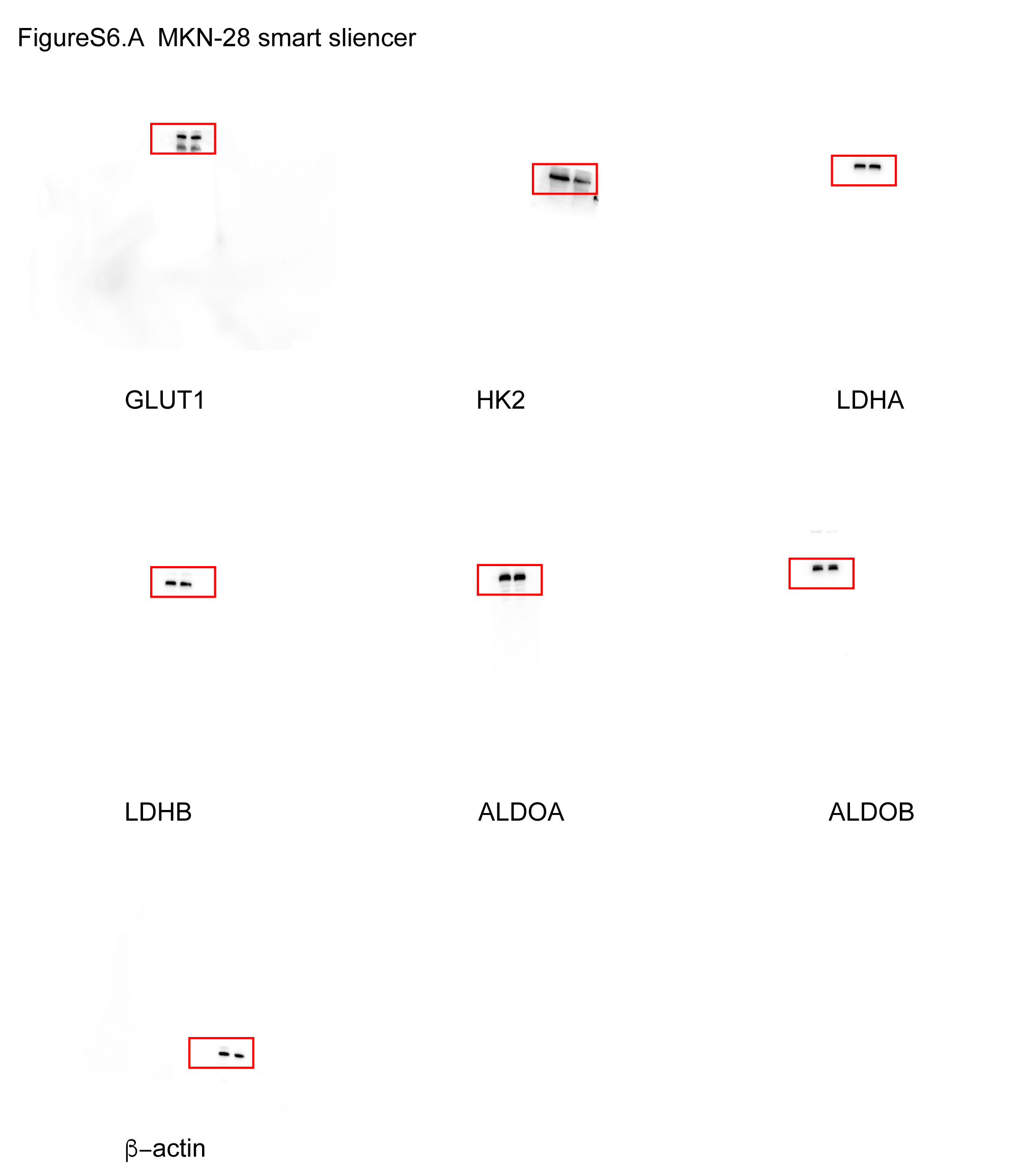

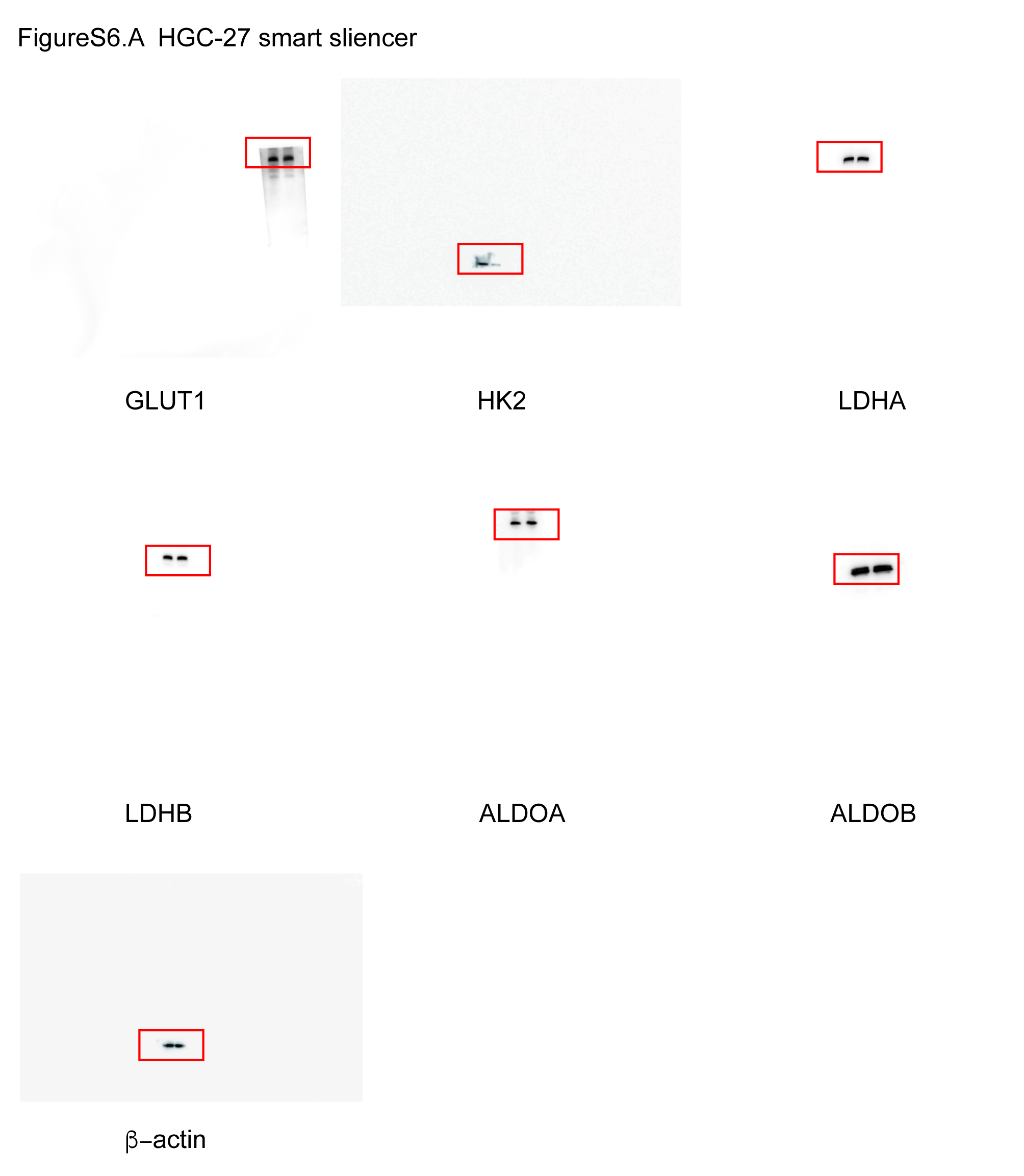

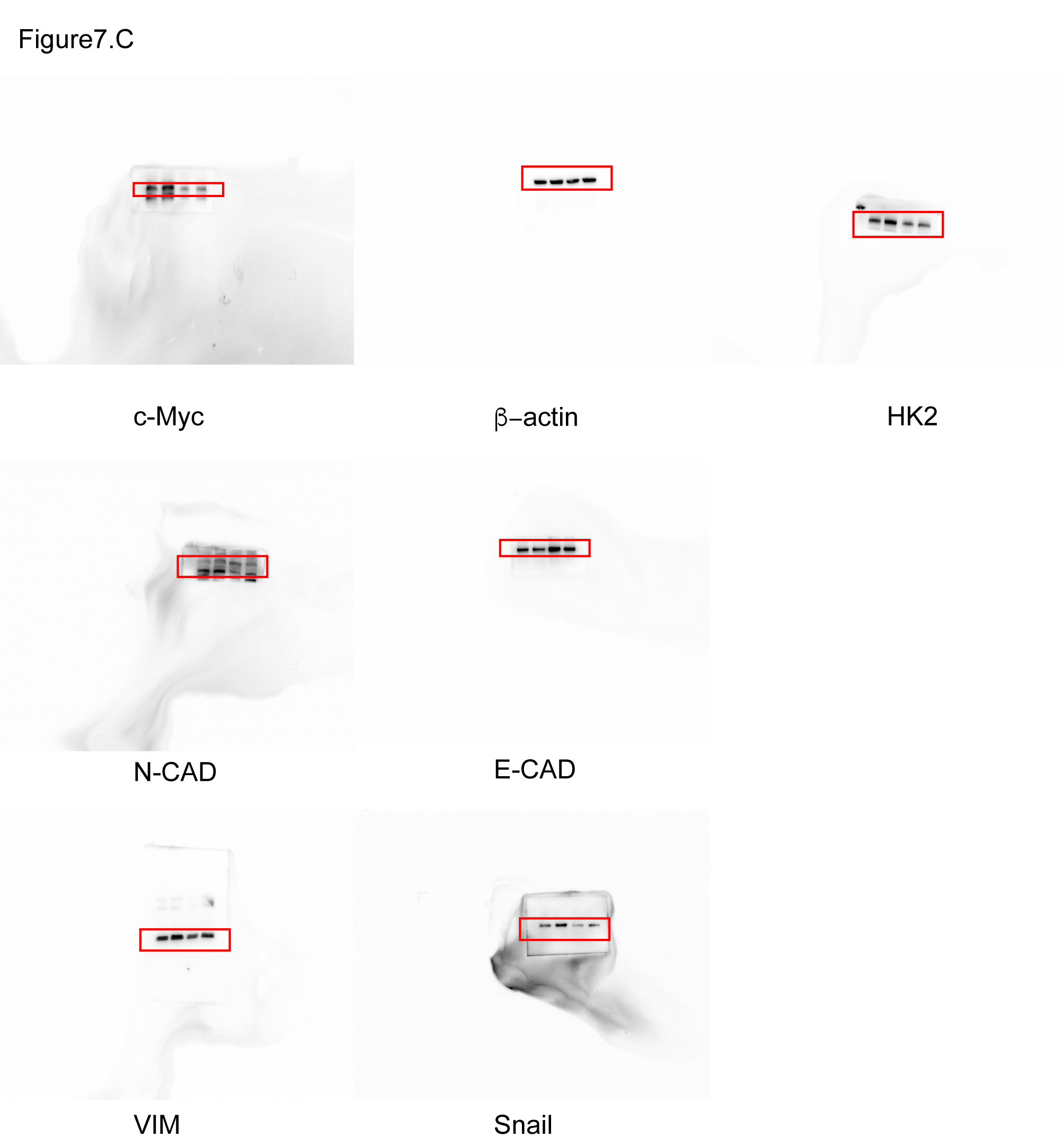

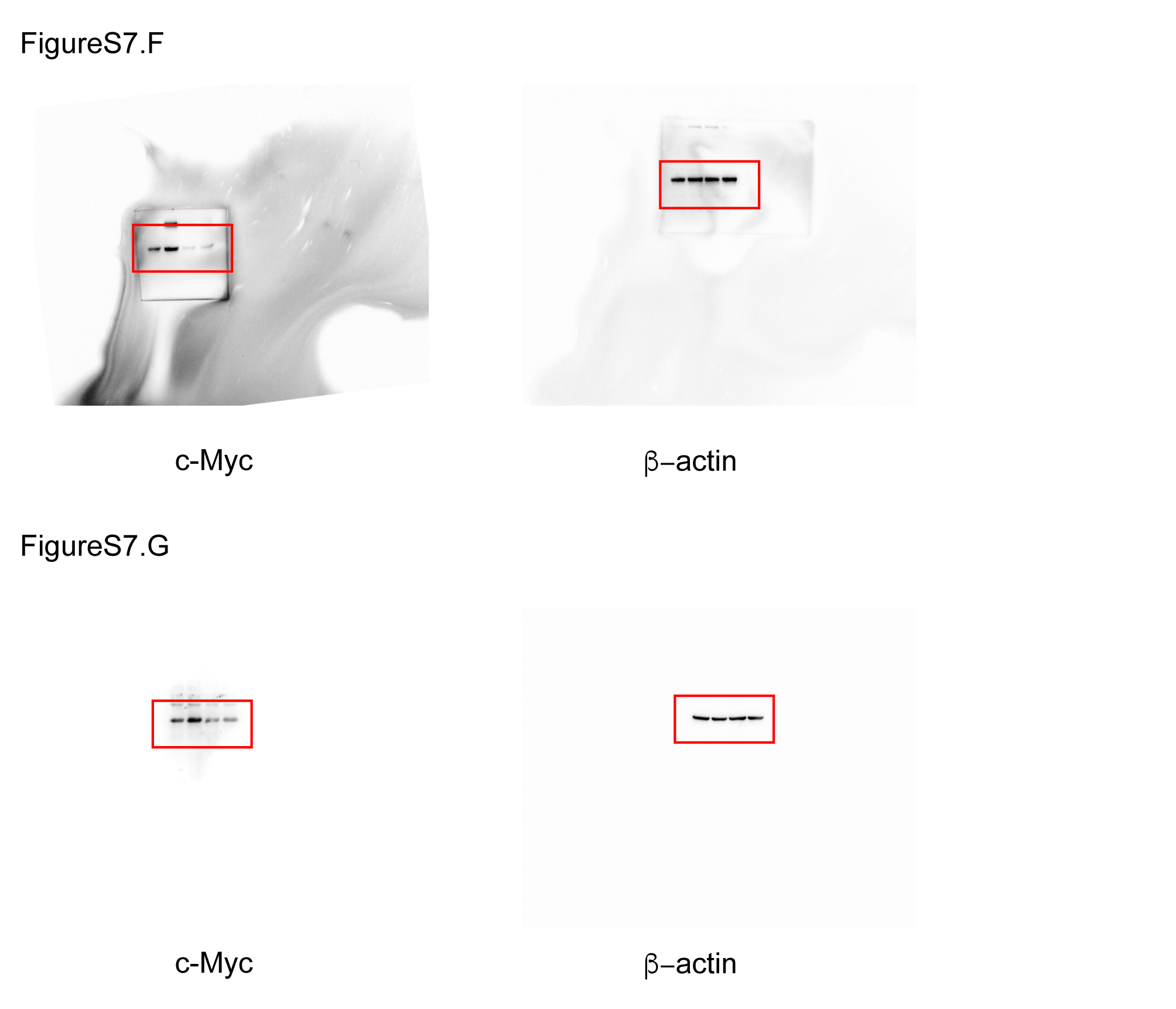

Supplement: Supplementary file 2 — original western blots [file 41419_2024_6607_MOESM2_ESM.docx]
